# The protective effects of gastrodin on neurological disorders: an update and future perspectives

**DOI:** 10.3389/fphar.2024.1494277

**Published:** 2024-12-24

**Authors:** Zhouying Shi, Yali Zhang, Yuhua Xiao, Zhoujing Shi, Xiaotong Wei, Bin Wang, Yue Yuan, Ping Li

**Affiliations:** ^1^ College of Traditional Chinese Medicine, Changchun University of Chinese Medicine, Changchun, China; ^2^ College of Basic Medicine, Changchun University of Chinese Medicine, Changchun, China; ^3^ College of Basic Medicine, Heilongjiang University of Chinese Medicine, Harbin, China; ^4^ College of Nursing, Changchun University of Chinese Medicine, Changchun, China

**Keywords:** gastrodin, neurological disorders, molecular mechanism, pharmacological effects, pharmacokinetics, drug delivery system

## Abstract

Neurological disorders are characterized by high mortality and disability rates. Furthermore, the burden associated with disability and mortality resulting from neurological disorders has been increasing at an alarming rate. Botanical drugs and their bioactive components have emerged as a prominent area of research, offering a promising avenue for developing novel alternatives for treating neurological diseases. Gastrodin is the principal active component derived from the traditional Chinese medicinal plant *Gastrodia elata* Blume (GEB). Existing literature reveals that gastrodin exerts various pharmacological protective actions against neurological disorders. This review aimed to collate novel literature on gastrodin for treating neurological disorders from Web of Science, PubMed, Embase and CNKI. The pharmacokinetics of gastrodin, its therapeutic role in neurological disorders, the main mechanisms of action and clinical application were addressed. Furthermore, a detailed overview of gastrodin drug delivery systems and physical enhancement methods was presented, offering invaluable insights into potential research and the extensive applications of gastrodin.

## 1 Introduction

Neurological disorders are the primary reason for disability and the second leading cause of mortality worldwide (Feigin et al., 2020). Smoking, alcohol consumption, and high intake of sugar and salt levels are important risk factors for the development of neurological disorders (Collaborators, 2019). The most common neurological disorders with high rates of mortality and disability are stroke, migraine, vascular dementia (VD), and Alzheimer’s disease (AD) (Feigin and Vos, 2019), followed by epilepsy, traumatic brain injury (TBI), Parkinson’s disease (PD), peripheral nerve injury (PNI), hypoxic-ischemic brain damage (HIBD), and others (Wei et al., 2023; Min et al., 2020; Liu B. et al., 2018). Currently, the treatment of neurological diseases is mainly focused on symptom management and delaying disease progression, rather than targeting the underlying pathological cause, and treatment with these drugs often has limited efficacy and side effects (Rahimi Darehbagh et al., 2024). Developing new drugs is a central objective of scientific research in this field.

Compared to modern pharmaceuticals, traditional herbal plants have been successfully used for centuries (Choi et al., 2011). The botanical drug GEB is a perennial plant. In the first century BC, GEB was documented as a superior Chinese medicine agent with a long history of use for treating stroke and epilepsy (Zhan et al., 2016). Modern research has extracted approximately 134 components from GEB, including phenols, polysaccharides, and glycosides (Gong et al., 2024). Gastrodin (GAS) is the principal active component due to its neuropharmacological properties (Kim et al., 2012), which extracted from GEB by reflux extraction, ultrasound-assisted extraction and microwave extraction (Teo et al., 2008). However, due to the low content of GAS in GEB, traditional isolation and purification methods are unable to obtain a high yield, high purity and low cost of GAS. Therefore, GAS used in the current research is mainly produced by chemical and biological synthesis (Dai et al., 2024). The research findings indicate that GAS can reduce inflammation and oxidative stress-induced neurological damage (Xiao et al., 2023). It has been widely used to treat neurological disorders (More et al., 2013; Jangra et al., 2022; Fan et al., 2024). Nevertheless, the extensive application of GAS and its molecular mechanism of action remain significant challenges due to its favorable hydrophilicity, low blood-brain barrier (BBB) permeability, and poor bioavailability (Illum, 2003; Huang et al., 2022; Wang et al., 2021).

A literature review revealed many studies examining the therapeutic potential of GAS in managing neurological disorders. The majority of these studies focused on central nervous system (CNS) disorders He et al. (2024); Liu B. et al. (2018), providing a review of the chemical structure, pharmacokinetics, and pharmacological effects of GAS (Dai et al., 2024). There is a lack of reviews on the studies of GAS in peripheral nervous system disorders, GAS delivery systems, and clinical studies of its monotherapy and combinations on neurological disorders. Consequently, this paper provided a comprehensive review of studies related to GAS in central and peripheral nervous system disorders. In addition, the paper discussed the GAS drug delivery system, physical enhancement methods, their primary mechanisms of action in neurological disorders and clinical application in great detail. Finally, we put forward suggestions regarding future research directions in light of the limitations of current research on GAS and the potential for drug development. Previous literature reviews of the neurological disorders of GAS treatment reported a lack of coverage of these aspects (Xiao et al., 2023; Liu Y. et al., 2018). This review article aimed to overcome these limitations and further contribute to the extensive research and applications of GAS.

## 2 Search strategy and selection criteria

### 2.1 Search strategy

Web of Science, PubMed, Embase and CNKI databases were searched for all relevant articles published in the English language from the database inception until 30 March 2024 using the following terms: “gastrodin”, “gastrodia elata Blume”, “neurological disorders”, “cerebrovascular disease”, “ischemic stroke”, “hemorrhagic stroke”, “vascular dementia”, “Parkinson’s disease ”, “Alzheimer’s disease”, “neurodegenerative disease”, “epilepsy”, “migraine”, “traumatic brain injury”, “peripheral nerve injury”, “hypoxic-ischemic brain damage”, “depression”, “nervous system tumours”, “pharmacokinetics”, “drug delivery systems”, “nanoparticles”, “polyurethane porous membranes”, “solid dispersions”, “*in situ* gelling systems”, “focused ultrasound”, “focused shock wave.”

### 2.2 Selection criteria

The inclusion criteria were as follows: GAS was the study subject; the diseases treated by GAS belonged to the neurological disorders category; and the study results involved the exploration of the mechanisms. The exclusion criteria were: unclear neurological diseases treated by GAS; unclear research object, research method, and mechanism of action in case reports and literature reviews; poor methodology, unreliable results or poor quality; repetitive publication and duplication of research content in the literature.

Two researchers (ZS and YZ) undertook an independent screening and analysis of all the literature during the collation process, thus ensuring the reliability of the study.

## 3 Pharmacokinetics of gastrodin

GAS is a phenolic glucoside active component with good hydrophilicity and the highest systemic exposure compared to other *G. elata* extracts (Dong et al., 2020). GAS is rapidly absorbed in the intestinal tract. A metabolic kinetics study of GAS in SD rats showed that the time to reach peak plasma concentration (T_max_) of GAS administered as a single dose of 100 mg/kg was 0.5 h in both the ingested and fasted states. However, the peak plasma concentration (C_max_) and the area under the curve (AUC) in the fasted state were approximately twice those observed in the ingested state (Jia et al., 2014). The half-life (t_1/2_) of fasting administration was markedly longer than feeding administration, suggesting that fasting may lead to better and faster GAS absorption (Jia et al., 2014). Following oral administration, GAS was rapidly absorbed into the blood and widely distributed throughout the organs and tissues. Moreover, Jiang et al. (2017) observed that GAS is predominantly distributed in the kidneys, with a concentration of 12,584.06 ng/g, followed by the heart (2034.08 ng/g), liver (1592.58 ng/g), and lung (1433.08 ng/g). The lowest distribution was observed in the brain and spleen, which is consistent with results reported by Lin et al. (2007), who observed that the C_max_ of GAS (100 mg/kg i. v.) in the brain and blood of SD rats was 1.4 and 533 μg/mL, respectively. This may be related to the good hydrophilicity and low BBB permeability of GAS (Ji et al., 2015).

GAS is rapidly absorbed and enters the bloodstream, where it undergoes a biotransformation process and is metabolized into various metabolites (Cheng and Deng, 2021). It has been reported that the urinary excretion rate of GAS reaches its peak at the 1-h mark following administration, with a cumulative excretion rate of 53% within 0–48 h, which suggests that urine is the primary route of excretion of GAS (Zhang et al., 2019). However, Jiang et al. (2017) examined the excretion of GAS in the urine and feces of SD rats following the administration of GEB powder (4 g/kg orally) and GEB extract (0.6 g/kg orally). The results showed that the renal excretion of GAS within 24 h of oral administration was <51.42% (bulk) and <18.13% (extract). The quantity excreted in feces was minimal. In addition, Nepal et al. (2019) confirmed that GAS is readily hydrolyzed by intestinal flora in the intestinal tract and rapidly converted to 4-HBA, which is significantly different from the pharmacokinetics of rats after antibiotic use. This indicates that the absorption and metabolism of GAS in the intestinal tract mainly depend on the intestinal microbiota. It may be concluded that renal and intestinal metabolism are the main metabolic pathways of GAS. Nevertheless, the quantity of GAS excreted in urine and feces is restricted, and GAS may be excreted via alternative metabolic pathways through conversion to other metabolites.

Recent research has found that combining botanical drugs may prolong the activation time of drugs and enhance the absorption of active components (Tang et al., 2022). The combination of *Ligusticum chuanxiong* with GEB represents a well-established approach to treating migraine (Wang et al., 2016). Tetramethylpyrazine (TMP) and ferulic acid (FA) represent the principal active components of *L. chuanxiong*. In their study, Mi et al. (2020a) used a migraine SD rat model of liver yang ascendant hyperactivity, where the C_max_ of SD rats treated with GAS (915 mg/kg. i. g.) alone was 132.95 μg/mL. The C_max_ of SD rats in the group treated with GAS (915 mg/kg. i. g.) in combination with TMP and FA was 314.33 μg/mL. Furthermore, the AUC, mean retention time (MRT), and t_1/2_ were noticeably lower in the combined treatment group than the GAS alone group. The metabolic kinetics of GAS in the brain tissue of migraine model rats exhibited a consistency with the results observed in blood. Also, the T_1/2_ increased with the rising TMP and FA levels (Mi et al., 2020b). The results suggest that adding TMP and FA enhances the permeability of the BBB for GAS, thereby facilitating its absorption and metabolism. Moreover, Mi et al. (2020a) confirmed that GAS and FA exerted bidirectional regulatory effects in the blood-stasis migraine model. It was observed that the T_1/2_, MRT, C_max,_ and AUC in the blood and brain interstitial fluid (BIF) of the SD rats in the group that received GAS exhibited a notable increase compared to the group that did not. Furthermore, the degree of TMP uptake in the blood and BIF was positively correlated with the concentration of GAS. These results suggest that GAS may markedly enhance the bioavailability and prolong the duration of FA by reducing BBB permeability (Mi et al., 2020a). The combination of GAS and other treatments has been demonstrated to reduce neuropathy and modulate pain pathways effectively (Ferrari et al., 2024). These findings offer insights into potentially novel approaches for treating neurological disorders.

## 4 Drug delivery system and physical enhancement methods

**FIGURE 1:**
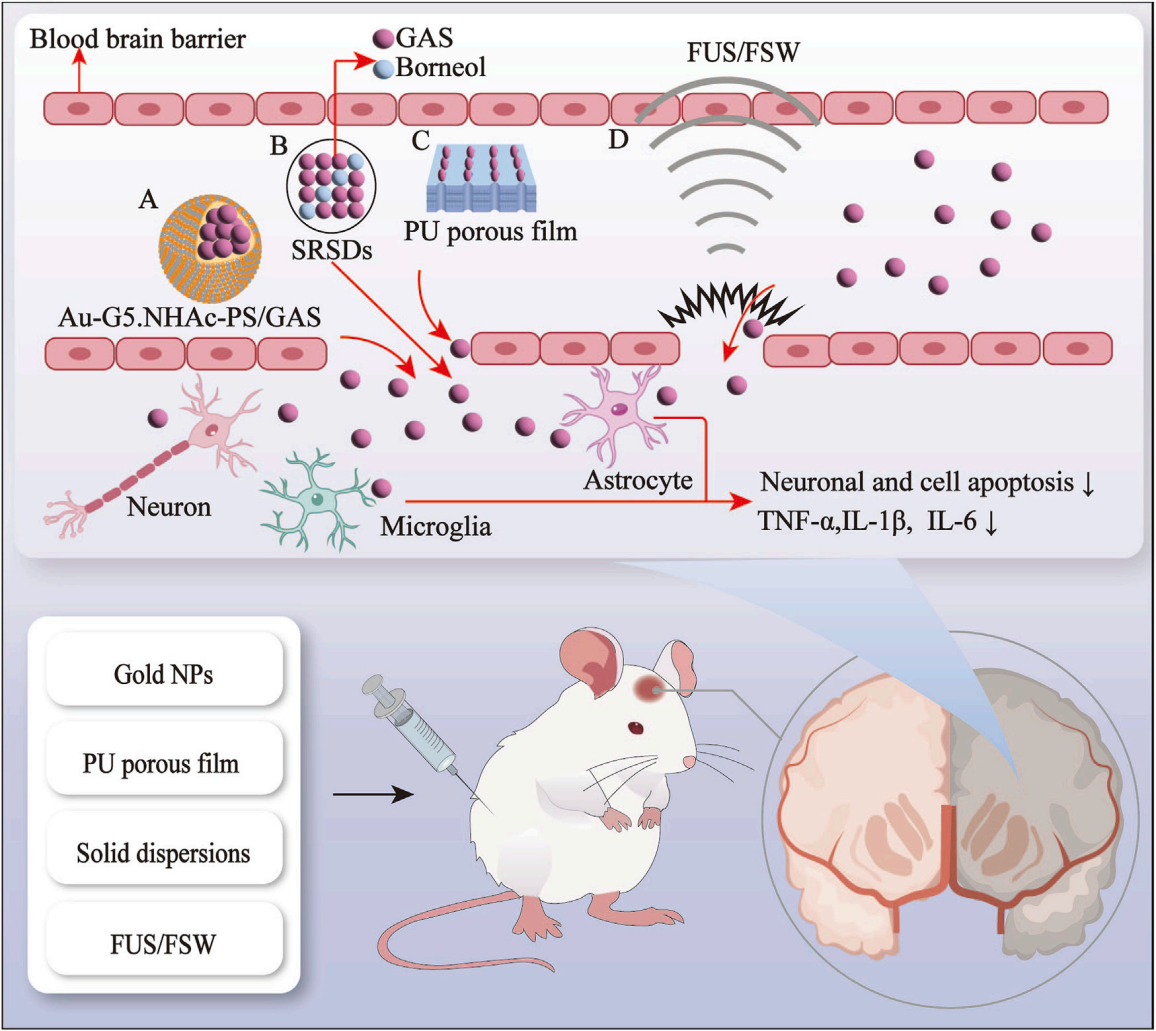
The gastrodin drug delivery system and physical enhancement methods via the blood-brain barrier route. A, gold nanopaticles; B, solid dispersions, C, polyurethane porous film; D, focused ultrasound and focused shockwave.

**FIGURE 2:**
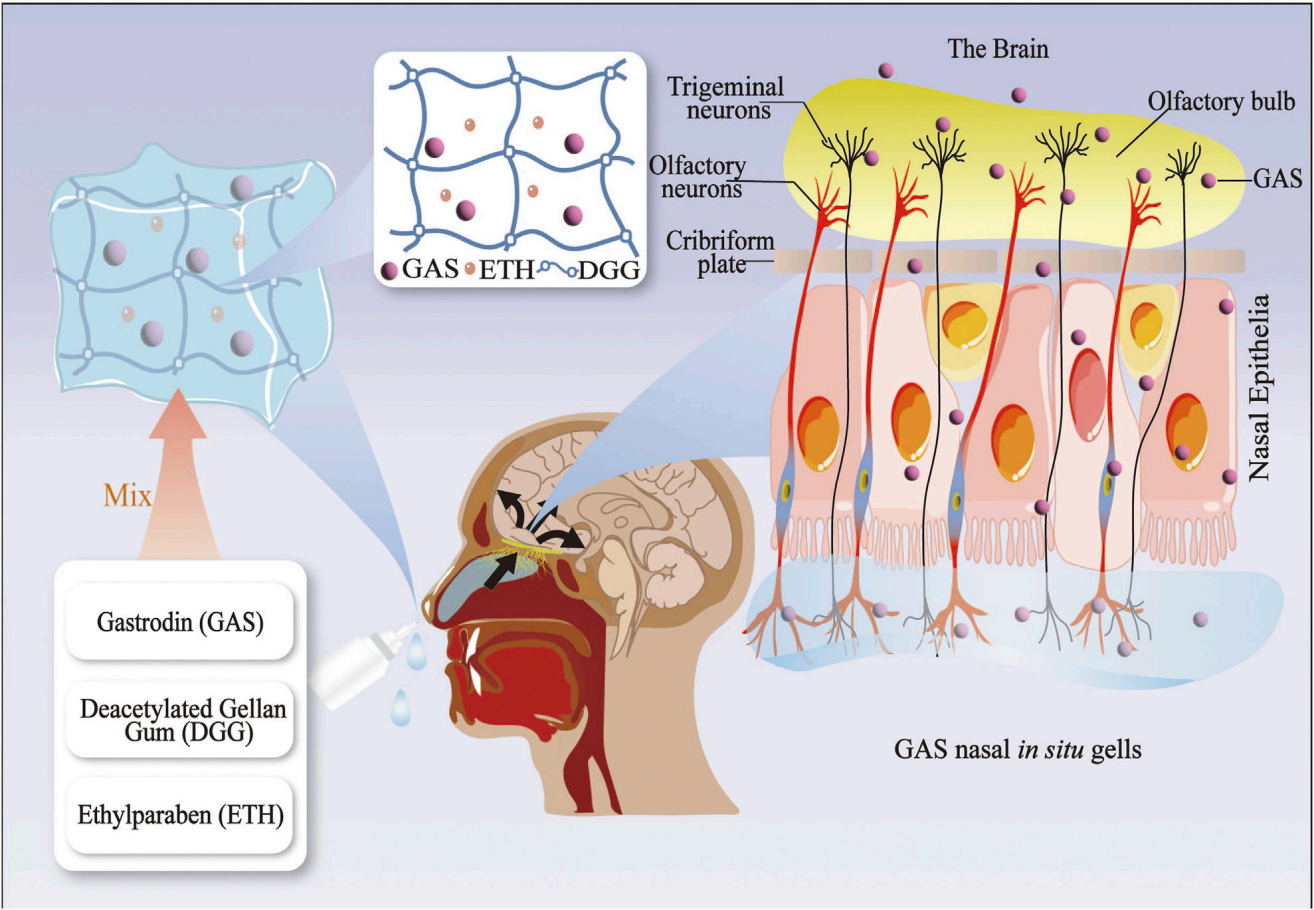
The gastrodin drug delivery system via the nose-brain route (GAS nasal *in situ* gelling systems).

The BBB represents a significant obstacle in treating neurological disorders, impeding the delivery of pharmaceutical agents into the brain (Zhang et al., 2021). Hydrophilicity of GAS severely affects the therapeutic efficacy and bioavailability of GAS for neurological disorders (Long et al., 2020). In recent decades, nanoscience, ultrasound science, and natural components have been gradually integrated into medical science research to develop safe, highly biocompatible, and bioavailable drug delivery systems. In light of the growing interest in drug delivery systems, various innovative systems based on GAS have been developed. These include gold nanoparticles (AuNPs), polyurethane (PU) porous film, solid dispersion (SD), and *in situ* gelling systems (ISGS) (Shen et al., 2024; Li et al., 2020; Cai et al., 2014). Furthermore, the combination of physical methods, including focused ultrasound (FUS) and focused shockwaves (FSW), with GAS also improves the delivery of GAS in the brain (Kung et al., 2021) (see Figures 1, 2; Table 1, 2).

**TABLE 1:** Improved properties of gastrodin drug delivery system and physical enhancement methods.

Delivery system	Improved properties	Refs.
Gold nanoparticles	Improved bioavailability; reduced cytotoxicity; facilitated cellular uptake; prolonged persistence in the blood circulation	Huang et al. (2022)
PU porous film	Improved hydrophilicity; excellent biocompatibility; maintains cells with more neurite extension concomitantly	Li et al. (2020)
Solid dispersions	Improved bioavailability; reduced stomach irritation; improved and maintained brain targeting	Cai et al. (2014)
*In situ* gelling systems	Improved bioavailability; enhanced analgesic and sedative effects	Cai et al. (2011)
Focused ultrasound	Increased permeability of blood-brain barrier; increased drug uptake; improved brain targeting	Wang et al. (2022), Luo et al. (2022), Wang et al. (2021)
Focused shockwave	Improved brain targeting; increased and prolonged circulation of drugs in the cerebrospinal fluid	Kung et al. (2021)

Refs., references; PU, Polyurethane.

**TABLE 2:** Gastrodin drug delivery system and physical enhancement methods studied in preclinical acute neurological disorders.

Delivery system	Disease	Experimental model	Targets	Main results	Refs
Gold nanoparticles	CIRI	*In vivo:* SD rats	*In vivo* and *In vitro*: NA	*In vivo and in vitro:* decreased the apoptosis rate and improved the therapeutic effects of GAS on CIRI.	Huang et al. (2022)
		*In vitro:* astrocytes/hypo-thalamic neurons			
PU porous film	PNI	*In vitro:* PC12 cells	*In vitro*: BDNF, GDNF↑	*In vitro:* promoted the regeneration, proliferation, and differentiation of nerve cells	Li et al. (2020)
Focused ultrasound	AD	*In vivo:* Kunming mice	*In vivo*: AQP4, BDNF, SYN, PSD-95↑	*In vivo:* alleviated memory deficit and neuropathology of the AD-like mouse model	Luo et al. (2022)
			Aβ, tau, p-tau↓		
Focused ultrasound	PD	*In vivo:* C57BL/6J mice	*In vivo*: TH, DAT, Bcl-2, BDNF, SYN, PSD-95↑	*In vivo:* strengthened the protective effect of GAS on dopaminergic neurons through reinforcing the anti-apoptotic activity and the expression of synaptic-related proteins	Wang et al. (2022)
			caspase-3↓		
Focused shockwave	Epilepsy	*In vivo:* SD rats	*In vivo*: SOD, CAT, GSH, T-AOC↑	*In vivo:* epileptiform discharges, inflammation, oxidative stress, and apoptosis were reduced	Kung et al. (2021)
			IL-1β, MDA↓		

Refs., references; CIRI, cerebral ischemia–reperfusion injury; NA, not applicable; GAS, gastrodin; PU, polyurethane; PNI, peripheral nerve injury; BDNF, brain-derived neurotrophic factor; GDNF, glial cell derived neurotrophic factor; AD, Alzheimer’s disease; AQP4, aquaporin-4; SYN, synaptophysin; PSD-95, postsynaptic density protein 95; Aβ, beta-amyloid; tau, tubulin associated unit; p-tau, phosphorylated tau; PD, Parkinson’s disease; TH, tyrosine hydroxylase; DAT, dopamine transporter; Bcl-2, B-cell lymphoma 2; SOD, superoxide dismutase; CAT, catalase; GSH, glutathione; T-AOC, total antioxidant capacity; IL-1β, interleukin-1β; MDA, malondialdehyde.

### 4.1 Drug delivery system

#### 4.1.1 Gold nanoparticles (AuNPs)

AuNPs are made of gold, which has safe and distinctive biomolecular characteristics (Lu et al., 2021). Currently, AuNPs are utilized in several biomedical applications such as drug delivery, cellular imaging, etc. (Arami et al., 2022; Betzer et al., 2017). In particular, these nanoparticles are employed extensively in diagnosing and treating neurodegenerative diseases. E.g., AuNPs can delay the development of Alzheimer’s disease (AD) by inducing the polarization of macrophages to the M2 phenotype and attenuating neuroinflammation (Aili et al., 2023). Additionally, AuNPs can target pathological proteins, facilitating early and precise diagnosis by enhancing protein signals (Chiang et al., 2024). Many protein detection sensors based on AuNPs have been developed, e.g., Tau and α-synuclein targeted AuNPs for diagnosing neurodegenerative disorders (Tapia-Arellano et al., 2024). Over the years, AuNPs have become increasingly important as potential tools for diagnosing and treating neurological diseases (Baez et al., 2021).

While AuNPs improve drug penetration across the BBB (Sokolova et al., 2020), the delivery of AuNPs into the brain is constrained by the BBB. Consequently, contemporary researchers have sought to enhance the functionalization of AuNPs to facilitate their delivery by modifying the surface of the AuNPs using carrier materials (Baez et al., 2021). GAS does not readily cross the BBB due to its hydrophilic nature. To enhance the efficacy of GAS on cerebral ischemia-reperfusion injury (CIRI), Huang et al. (2022) employed the physical embedding method to load GAS into a carrier material encapsulated with AuNPs, resulting in the preparation of the GAS-loaded drug delivery system Au-G5. NHAc-PS/GAS with excellent biocompatibility and sustained drug release capabilities. Au-G5. NHAc-PS/GAS significantly reduced and downregulated the production of pro-inflammatory factors compared to GAS alone. The nano drug-carrying system enhanced the penetration ability of the BBB of *Aspergillus* and significantly improved bioavailability (Huang et al., 2022).

#### 4.1.2 Polyurethane porous film

Polyurethane (PU) is a polymer with favorable properties, including mechanical properties, biodegradability, and biocompatibility, particularly applicable to biomedical applications (Wendels and Averous, 2021). Due to their straightforward processing, extensive availability, and low cost (Uscategui et al., 2019), polyurethanes have been developed into membranes, scaffolds, and gels for pharmaceutical use Arshad et al. (2024). The development of multifunctional membranes utilizing polymers such as polyurethanes has been shown to enhance protein loading capacity and regulate the release of nerve growth factor (Uz et al., 2017).

Several researchers have designed a novel GAS-crosslinking PU elastomer that enhances cellular activity by using the ability of polyurethane to modulate nerve growth factor release. However, this elastomer cannot differentiate nerves (Li et al., 2018). The high interconnectivity and interconnected porous film structure of the material facilitate peripheral nerve regeneration by improving oxygen and protein circulation (Hsu and Ni, 2009). Li et al. (2020) developed an elastomeric PU porous film functionalized with GAS, which has been demonstrated to promote peripheral nerve regeneration. This was achieved by combining GAS and PU porous film, building upon previous research on GAS-crosslinking PU elastomers, which improved the bioavailability of GAS. The potential of PU membranes in treating peripheral nerve injury is significant. However, there is a paucity of clinical or experimental studies on using polyurethane porous membranes in peripheral nervous system diseases or nerve injury-related neurological diseases. Future research should investigate the efficacy of polyurethane porous membranes in treating neurological disorders.

#### 4.1.3 Solid dispersion (SD)

SD is the combination of hydrophobic drugs with a hydrophilic carrier, which increases the solubility of the drug by changing its state (Tran and Park, 2021). The T_1/2_, AUC_0-t_, and AUC_0-α_ of andrographolide (50 mg/kg) in the plasma of Wistar rats were found to be 2.6 h, 532 and 608 ng h/mL, respectively. However, when the SD technique was employed, these values increased to 4.0 h, 653 ng/h, and 867 ng/h/mL, respectively (Assim et al., 2024). SD technology significantly reduces the drug clearance of andrographolide. Previous studies have also demonstrated that SDs enhance drug bioavailability by increasing water solubility and maintaining drug release (Gala et al., 2020).

Cai et al. (2014) used SD technology to prepare sustained-release SDs (SRSDs) for co-loading GAS and borneol. Their findings demonstrated that SRSDs markedly diminished gastric irritation, enhanced bioavailability, and sustained the adequate oral brain-targeted drug delivery capacity of GAS compared to oral GAS alone. Previous studies have demonstrated that the combination of GAS and borneol enhances the absorption of GAS via oral administration and improves the brain targeting of GAS (Yao et al., 2020). SDs have a beneficial impact on both hydrophobic and hydrophilic drugs. In particular, they enhance the absorption of hydrophilic drugs, such as GAS, while reducing adverse drug reactions, thus expanding the potential applications of drugs for neurological diseases.

#### 4.1.4 *In Situ* gelling systems

Under normal temperature conditions *in vitro*, the *in situ* gels exist as a liquid and change to a gel state when stimulated by different factors *in vivo* (Kolawole and Cook, 2023). *In situ* gels prolong drug retention time, improve bioavailability, and control release profiles at specific body sites (Agrawal et al., 2020). *In situ* gel formulations were initially developed for topical administration. They can be delivered to various parts of the body via the oral, ocular, nasal, and vaginal routes, where they are used to treat oral, ocular, nasal, and vaginal diseases, respectively (Kolawole and Cook, 2023).

Some researchers combined the GAS and nasal *in situ* gel into a GAS nasal ISGS, finding that a GAS (50 mg/kg) nasal *in situ* gel preparation had the same analgesic effect on Kunming strain mice as an oral GAS (100 mg/kg) preparation. Furthermore, mice in the nasal *in situ* gel preparation group had a significantly longer sleep duration than mice in the oral solution group after receiving the same dose of GAS. The nasal ISGS enhanced the bioavailability and brain targeting of GAS while also improving its analgesic and sedative efficacy (Cai et al., 2011). Recent research has corroborated the efficacy of nasal ISGS in treating neurological disorders. This approach improves drug bioavailability by circumventing the BBB through cellular and trans-neural pathways, facilitating direct drug delivery to the brain (Samaridou et al., 2020). At present, the nasal route of drug administration is employed as an alternative means of delivering drugs to the CNS (Patharapankal et al., 2023). Nevertheless, there is a paucity of research on the utilization of nasal ISGS in the context of neurological disorders, whereas there is more literature on ocular ISGS in relation to the optic nerve (Al-Kinani et al., 2018). Accordingly, developing an ophthalmic ISGS based on the eye-brain pathway may be feasible for potential future applications in treating neurological disorders.

### 4.2 Physical enhancement methods

#### 4.2.1 Focused ultrasound

The initial application of medical ultrasound in neurology was primarily focused on detecting the brain through Doppler ultrasound diagnosis. Highly focused ultrasound can stimulate a discrete region of the brain, facilitating independent navigation to accurately localize a specific brain area and regulate brain activity (Beisteiner et al., 2023), which is employed for highly focused tissue ablation and clinical neuromodulation brain stimulation. Besides, it has been shown that focused ultrasound can target focal BBB openings and reversibly disclose the localized openings, which are approximately 1 cm^3^ in volume, without causing damage to brain tissue (Huang et al., 2017). The FUS-mediated opening of the BBB has been increasingly recognized as a potential enhancement method for treating brain diseases (Wang et al., 2022). The combination of FUS and microbubble drug delivery has been demonstrated to facilitate highly targeted delivery of drugs to the brain, opening the BBB and reducing apoptosis (Wang et al., 2021). Research has also shown that combining FUS with intravenous microbubble administration offers significant advantages in treating neurological disorders, particularly neurodegenerative diseases (Gasca-Salas et al., 2021).

Luo et al. (2022) demonstrated that FUS-mediated GAS (100 mg/kg) was more efficacious than the same dose of GAS in ameliorating memory in an amyloid-beta (Aβ)_1–42_ peptide-induced AD mouse model. Another study demonstrated that the intervention of FUS significantly augmented the content of GAS in the sonicated hemisphere of a Parkinson’s mouse model and raised the expression of anti-apoptotic and synaptic-related proteins (Wang et al., 2022). The combined application of FUS has been demonstrated to improve the bioavailability and brain targeting of GAS, thereby providing a novel drug delivery route for the effective treatment of neurological diseases. However, the therapeutic effects of distinct, focused ultrasound frequencies on disparate neurological disorders may also vary (Chen et al., 2022; Bachu et al., 2021). Hence, the optimal frequency and safety value of focused ultrasound in relation to adaptive diseases warrant further investigation.

#### 4.2.2 Focused shockwave

A shock wave is a specific type of sound wave classified according to the characteristics of shock wave energy focusing that can be divided into three main categories, i.e., FSW, radiating shock wave, and plane shock wave. Compared to the latter two, FSWs are more complex to operate; however, they offer precise positioning characteristics and the capacity to treat deep-seated lesions. Similarly to FUS, FSW can open the BBB in a controlled and reversible manner (Kung et al., 2020).

Kung et al. (2021) investigated the effect of FSW on the blood-cerebrospinal fluid barrier (BCB). Their findings revealed that FSW-mediated opening of the BCB substantially increased the level of systemically-administered GAS in the cerebrospinal fluid, accompanied by a prolonged retention time of GAS. Simultaneously, epileptiform discharges, neuroinflammatory responses, and apoptosis in the brain were diminished by the FSW-GAS treatment (Kung et al., 2021), which may be related to the fact that FSW noninvasively and selectively induces the opening of the cerebrospinal fluid barrier in specific brain regions enriched with choroid plexus by disrupting or tightly connecting open channels through acoustic pressure (Kung et al., 2022; Liao et al., 2020). Notably, the choroid plexus is not the same as the capillaries of the BBB. The vessel wall is more permeable, and the blood flow in the choroid plexus is greater. Therefore, compared with the FUS-induced opening of the BBB, FSW facilitates the delivery of drugs to a greater extent and with greater speed by opening the BCB (Kung et al., 2022). Nevertheless, the literature on focused shock waves in neurological disorders primarily includes clinical studies on post-stroke spasticity diseases (Starosta et al., 2024). There is a notable dearth of research on other neurological-related diseases and drug delivery, so further investigation and improvement are required.

In order to promote the bioavailability and the efficacy of treatment with GAS, a range of drug delivery systems and physical enhancement methods for GAS have been investigated to increase its BBB permeability, bioavailability, brain targeting, drug release rate, and *in vivo* retention time. Nevertheless, these novel drug delivery systems and physical enhancement methods have yet to be widely adopted in clinical practice, and there is a limited number of preclinical studies. Further studies are warranted to evaluate their safety and efficacy.

## 5 Neuroprotective effects of GAS on neurological disorders

### 5.1 Effects of GAS on cerebrovascular diseases

#### 5.1.1 Anti-ischemic stroke (IS) effects

IS is the most prevalent cerebrovascular disease (Hu et al., 2017) and the primary cause of disability and mortality in adults in China (Wu et al., 2019). The primary pathological characteristic of IS is damage to brain tissue, which is caused by neuronal apoptosis and inflammation (Tuo et al., 2022).

In the IS model, GAS might be a novel method to attenuate nerve damage, mainly because GAS inhibits the assembly of the nod-like receptor protein (NLRP) 3 inflammasome and the expression of oxidation factors in astrocytes. Sui et al. (2019) used different concentrations of GAS to treat OGD-exposed TNA2 astrocytes, finding that GAS effectively inhibited the expression of NLRP inflammatory vesicles and inflammatory factors in astrocytes, especially when the concentration of GAS was 20 μM. Also, GAS inhibited the expression levels of NLRP3 and NLRC4 most significantly. The study also found that GAS effectively inhibits signal transducer and activator of transcription (STAT3) in astrocytes of the middle cerebral artery occlusion (MCAO)-induced IS rat model and concluded that GAS suppresses NLRP3 inflammatory vesicles by restraining the NF-κB pathway, thereby attenuating the inflammatory response of IS. Using the same model, Peng et al. (2015) discovered that GAS (100 mg/kg) significantly activates the protein kinase B (AKT)/nuclear factor erythroid 2-related factor 2 (Nrf2) pathway, thereby attenuating neurological injury by inhibiting interleukin (IL)-1β, tumor necrosis factor (TNF)-α and increasing the expression of oxidative factors such as superoxide dismutase (SOD) and heme oxygenase 1 (HO-1). Furthermore, a transient MCAO-induced stroke model also proved that GAS could promote the expression of antioxidant genes in astrocytes by up-regulating Nrf2 and nuclear translocation, as well as significant attenuation of the massive neuronal damage caused by Zn^2+^ overload in late stroke (Luo et al., 2018). It is suggested that the inhibition of GAS on neuronal apoptosis could be related to Nrf2-mediated inflammation and oxidative stress. Another bilateral common carotid artery occlusion (BBCAO)-induced IS model found that GAS facilitated hippocampal neuronal regeneration via the phosphodiesterase 9 (PDE9)-cyclic guanosine monophosphate (cGMP)-protein kinase G (PKG) pathway, demonstrating the neuroprotective effect of GAS on IS(Xiao et al., 2021).

**FIGURE 3:**
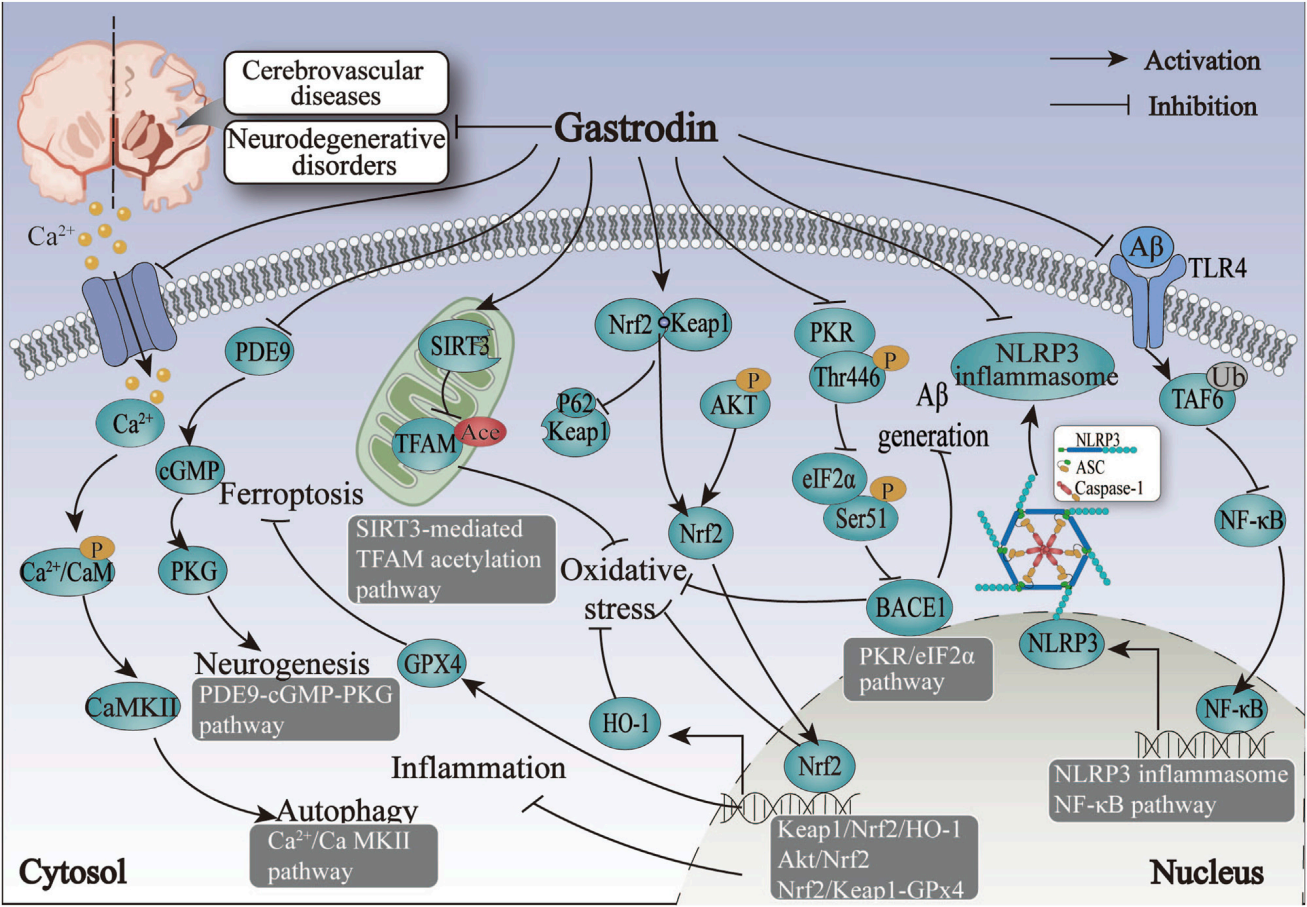
The therapeutic mechanism and critical pathways of gastrodin against cerebrovascular diseases and neurodegenerative disorders.

The studies above indicate that regulating nervous function may represent a primary mechanism for treating IS with GAS. GAS may exert its neuroprotective effects in IS by modulating the NF-κB, NLRP3, AKT/Nrf2 inflammatory and oxidative stress pathways and promoting neural regeneration by neural-related signaling pathways such as PDE9-cGMP-PKG pathways (Figure 3; Table 3).

**TABLE 3:** Summary of the targets/pathways/mechanisms and effects of gastrodin on cerebrovascular diseases.

Disease	Inducer	Experimental model	Dose	Targets/mechanisms	Effects	Refs.
IS	*In vivo:* NA	*In vivo:* C57BL/6J mice	*In vivo*: 50, 100 mg/kg (i.p. injection)	*In vivo:* cGMP, PKG, BDNF↑	Promotes hippocampal neuronal regeneration and attenuated neurological damage after cerebral ischemia via the PDE9-cGMP-PKG pathway	Xiao et al. (2021)
				activity of PDE9↓		
	*In vitro:* NA	*In vitro:* Neural stem cells	*In vitro*: 0.01, 0.1, 1 μM/L	*In vitro:* cGMP, PKG, BDNF↑		
				PDE9↓		
IS	*In vivo:* NA	*In vivo:* SD rats	*In vivo:* NA	*In vivo:* protein expression levels of NLRP3, NLRC4, IL-18, caspase-1, p-STAT3↓	Decreases the expression of NLRP3 and NLRC4 inflammasome via inhibition of STAT3 and NF-κB signaling pathway	Sui et al. (2019)
	*In vitro:* NA	*In vitro:* TNA2 astrocytes	*In vitro:* 10, 20, 50, 100 μM	*In vitro:* mRNA and protein expression of NLRP3, NLRC4, IL-18, caspase-1↓		
				protein expression of p-STAT3↓		
IS	*In vivo:* NA	*In vivo:* SD rats	*In vivo:* 20, 40, 80 mg/kg (i.p. injection)	*In vivo:* PH, pCO_2_, Glucose↓	Inhibits Zn^2+^-induced astrocytic cell death by inhibiting the inductions of p67 and PARP-1	Luo et al. (2018)
				pO_2_↑		
	*In vitro:* zinc sulfate	*In vitro:* C6 astroglial cells	*In vitro:* 50, 100, 250 μM	*In vitro:* Nrf2, HO-1, GCLM, NAD↑		
				ROS, p67, PARP-1, PAR↓		
IS	*In vivo:* NA	*In vivo:* C57BL/6J mice	*In vivo:*10, 50, 100 mg/kg (i.p. injection)	*In vivo:* caspase-3, Bax↓, MDA↓, TNF-α, IL-1β↓	Activates the Akt/Nrf2 pathway and exerts neuroprotective effects against cerebral ischemia through anti-inflammatory, anti-oxidative, and anti-apoptotic pathways	Peng et al. (2015)
				Bcl-2, SOD, Akt, p-Akt, Nrf2, HO-1↑		
HS	*In vivo:* Collagenase IV	*In vivo:* SD rats	*In vivo:*100 mg/kg (i.p. injection)	*In vivo:* ROS, 8-OHDG, 3-NT, MDA↓	Activates Keap1/Nrf2/HO-1 signaling pathway to reduce inflammatory and oxidative factors and inhibit neuronal apoptosis.	Liu et al. (2020)
				Keap1, Nrf2, HO-1, Bcl-2, GSH-Px, SOD↑		
				Bax, caspase-3, caspase-9↓		
	*In vitro:* Hematoma lysate	*In vitro:* Primary cortical neuron	*In vitro*: 0, 10, 100, 300 μM	*In vitro:* cell viability↑, cell apoptosis↓		
HS	*In vitro:* NA	*In vivo:* SD rats	*In vivo:* 100 mg/kg (i.p. injection)	*In vivo:* Nrf2, HO-1, SOD, p-Akt, Bcl-2↑	Inhibits microglia and astrocyte activation, oxidative stress, and neuronal apoptosis by reducing glutamate excess-mediated neurotoxicity.	Wang et al. (2019)
				IL-1β, TNF-α, GFAP, MDA, 3-NT, 8-OHDG, Bax↓		
				concentration of Glu, Ca^2+^↓		
VD	*In vivo:* NA	*In vivo:* SD rats	*In vivo*: 15, 30, 60 mg/kg (i.g.)	*In vivo:* p62, Bcl-2↑	Attenuates Aβ deposition, inhibiting autophagy and apoptosis via P38 MAPK signaling pathway.	Liu et al., 2018
				p-P38 MAPK, Bax, Beclin-1, LC3-II, Aβ_1-40/42_, APP, BACE1↓		
VD	*In vivo:* NA	*In vivo:* SD rats	*In vivo*: 25, 50 mg/kg (i.p. injection)	*In vivo:* LC3, p62 and caspase-3↓, p-CaMKIIα↓,	Attenuates autophagic flux dysfunction by inhibiting the Ca^2+^/CaMKII signaling pathway to ameliorate cognitive impairment in VD.	Chen et al. (2021)
				LAMP-2↑		
	*In vitro:* CoCl_2_ 200 μM	*In vitro:* HT22 cells	*In vitro:* 200 μM	*In vitro:* LC3-II, LC3, p62, p-p62, Ca^2+^, p-CaMKIIα↓		
				LAMP-2↑		
VD	*In vivo and In vitro:* NA	*In vivo:* SD rats	*In vivo:* 25, 50 mg/kg (i.p. injection)	*In vivo and In vitro:* GPx4, Nrf2, GSH↑	Inhibits ferroptosis in hippocampal neurons by activating the Nrf2/Keap1-GPx4 signaling pathway	Li et al. (2022)
		*In vitro:* HT22 cells	*In vitro:* 25, 50, 100 μmol/L			
				Fe^2+^, MDA, COX2, Keap1↓		
VD	*In vivo:* NA	*In vivo:* SD rats	*In vivo:* 25, 50 mg/kg (i.g.)	*In vivo:* Aβ_1-42_, p-tau396, p-tau217↓	Enhances neuronal energy metabolism and improves neuronal mitochondrial function	Wu et al. (2023)
	*In vitro:* NA	*In vivo:* SD rats	*In vitro:* 12.5, 25, 37.5, 50, 75, 100 μM	*In vitro:* cell viability↑, mitochondrial dysfunction↓		
VD	*In vivo and In vitro:* NA	*In vivo:* SD rats	*In vivo:* 20, 50 mg/kg (i.p. injection)	*In vivo* and *In vitro:* The contents of ATP, NADH, NDUFB8, SDHB, UQCRC2, MTCO1, ATP5A, GSH↑	Alleviates mitochondrial dysfunction by attenuating the SIRT3-mediated TFAM acetylation pathway and increasing the production of ATP, GSH, and SOD.	Chen et al. (2024)
		*In vitro:* HT22 cells	*In vitro*: 0, 50, 100, 200 μM	SOD, SIRT3, TFAM, Mfn1/2, HO-1, Prdx1↑		
				ROS, P53, P21, P16, Fis1, DRP1↓		

IS, ischemic stroke; NA, not applicable; cGMP, cyclic guanosine mono phosphate; PKG, protein kinase G; BDNF, brain-derived neurotrophic factor; PDE9, Phosphodiesterase 9; SD, Sprague-Dawley; NLRP, nod-like receptor protein; IL, interleukin; STAT3, Signal transducer and activator of transcription 3; Nrf2, nuclear factor erythroid 2-related factor 2; HO-1, heme oxygenase-1; GCLM, Glutamate-cysteine ligase modifier; NAD, nicotinamide adenine dinucleotide; ROS, reactive oxygen species; PARP, poly ADP-ribose polymerase; MDA, malondialdehyde; TNF, tumor necrosis factor; SOD, superoxide dismutase; HS, hemorrhagic stroke; Akt, protein kinase B; GFAP, glial fibrillary acidic protein; 3-NT, 3-nitrotyrosine; Bcl-2, B-cell lymphoma-2; Bax, Bcl-2-associated X protein; 8-OHDG, 8-hydroxydeoxyguanosine; VD, vascular dementia; MAPK, mitogen-activated protein kinase; LC3-II, light chain 3-II; Aβ, amyloid β-protein; GSH-Px, antioxidant enzymes glutathione peroxidase; APP, amyloid precursor protein; BACE1, β-site APP, cleaving enzyme 1; CaMKIIα, Ca^2+^-calmodulin stimulated protein kinase IIα; LAMP, lysosomal-associated membrane protein; GSH, glutathione; GPx4, glutathione peroxidase 4; COX2, cyclooxygenase 2; ATP, adenosine triphosphate; NDUFB8, Ubiqui none Oxidoreductase Subunit B8; SDHB, succinate dehydrogenase complex iron sulfur subunit B; UQCRC2, ubiquinol-cytochrome c reductase core protein 2; MTCO1, mitochondrially encoded, cytochrome coxidase subunit 1; TFAM, transcription factor A; Mfn1/2, Mitofusin 1/2; HO-1, heme oxygenase-1; Prdx, Peroxiredoxin; Fis1, Fission 1; DRP1, dynamin-related protein1.

#### 5.1.2 Anti-hemorrhagic stroke (HS) effects

HS, including cerebral hemorrhage and subarachnoid hemorrhage (Ohashi et al., 2023), is a subtype of stroke with a high mortality and morbidity rate (Keep et al., 2012). The principal pathological mechanisms underlying the secondary injury following HS are inflammation and oxidative stress (Aronowski and Zhao, 2011).

Liu et al. (2020) demonstrated that GAS markedly influenced apoptotic factors and impeded neuronal apoptosis by reducing the expression of TNF-α, IL-1β, malondialdehyde (MDA), 8-hydroxydeoxyguanosine and 3-nitrotyrosine in the vicinity of hematoma following a collagenase-induced HS model in rats with HS. The inhibition of inflammatory and oxidative factors might be associated with the Kelch-like ECH-associated protein 1 (Keap1)/Nrf2/HO-1 pathway (Liu et al., 2020). Furthermore, Wang et al. (2019) proved that GAS (100 mg/kg) reduces glutamate and intracellular Ca^2+^ levels, increases Nrf2, HO-1, and Akt phosphorylation expression, and prevents microglia activation, neuronal apoptosis, and oxidative stress by reducing glutamate excess-mediated neurotoxicity in a rat model of subarachnoid hemorrhage (Li et al., 2011). The maintenance of glutamate homeostasis has been shown to protect against nerve damage caused by subarachnoid hemorrhage.

The protective mechanism of GAS against HS revolves around the processes of inflammation and oxidative stress, primarily through the activation of the Nrf2-mediated Keap1/Nrf2/HO-1 pathway, thus suggesting that Nrf2 may be the target of action of GAS in the treatment of HS (Figure 3; Table 3).

#### 5.1.3 Anti-vascular dementia (VD) effects

VD has become the second most prevalent form of dementia, following AD (Ismail et al., 2020). Most patients with VD also have the pathology of amyloid plaque formation and diffuse accumulation of neurofibrillary tangles in AD (van der Flier et al., 2018). Aβ has a significant role in the formation of amyloid plaque.

Chen et al. (2024) demonstrated that GAS (25 and 50 mg/kg, gavage) modulates the sirtuin 3 (Sirt3)-mediated transcription factor acetylation pathway and enhances the generation of adenosine triphosphate, superoxide dismutase (SOD), and glutathione (GSH), thus mitigating BBCAO-induced mitochondrial dysfunction in VD. This molecular mechanism may be related to the reduction of Aβ accumulation. The results of another bilateral common carotid artery ligation (2-VO) model of VD corroborated the significance of Aβ in VD, revealing that the same dose of GAS was efficacious in mitigating neuronal damage in VD rats by inhibiting Aβ production, lowering Aβ_1-40/42_ levels and activating tubulin associated unit (tau). This was also demonstrated *in vitro*, where GAS attenuated mitochondrial respiratory depression and metabolic dysfunction in H_2_O_2_-induced HT-22 cells (Wu et al., 2023). Furthermore, Liu B. et al. (2018) found that oral administration of GAS inhibited autophagy and apoptosis in neurons of a VD rat model by modulating the deposition of Aβ protein and inhibiting the expression of autophagy-associated proteins Beclin-1 and Light Chain 3 (LC3)-II, as well as the activity of apoptotic factors b-cell lymphoma (Bcl)-2 and p-P38 mitogen-activated protein kinase (MAPK). In another autophagy study (Chen et al., 2021), the investigators administered a 200 μM dose of GAS to H_2_O_2_-induced HT-22 cells, finding that GAS markedly diminished extracellular Ca^2+^influx in HT-22 cells, inhibited the generation of autophagic vesicles, facilitated their degradation, and sustained the equilibrium of autophagic flux via the Ca^2+^/Ca^2+^-calmodulin-stimulated protein kinase II (CaMKII) pathway. On the other hand, Li et al. (2022) found that GAS mitigated cognitive deficits in VD rats by inhibiting ferroptosis and enhancing antioxidant capacity via the activation of the Nrf2/Keap1-glutathione peroxidase 4 (GPx4) pathway.

The above summary demonstrates that regulating Aβ-related proteins by GAS is pivotal in alleviating ischemic hypoxia-induced VD. The alleviation of cognitive deficits after VD by GAS through mitochondrial metabolic disorders, autophagy, and the ferroptosis pathway offers promising avenues for the treatment of neurological disorders with cognitive dysfunction (Figure 3; Table 3).

### 5.2 Effects of GAS on neurodegenerative disorders

#### 5.2.1 Improvement of Alzheimer’s disease (AD)

AD is a common progressive neurodegenerative disorder. The accumulation of Aβ has been identified as a pathogenic factor of AD, which produces oxidative stress and induces neuroinflammation, thus exerting a considerable influence on the pathogenesis of AD (Zhang et al., 2023).

In their *in vitro* study, Zhao et al. (2012) administered dosages ranging from 1 to 100 μM of GAS to hippocampal neurons with Aβ_1-42_-induced, finding that GAS, particularly at 30 μM, markedly elevated the levels of catalase (CAT), and SOD and regulated active oxygen species, thereby reducing neuronal death. Meanwhile, they also found that GAS can mitigate Aβ-induced oxidative damage in neurons by modulating the ERK1/2-Nrf2 pathway. *In vivo* studies confirmed that GAS (60 mg/kg, gavage) was observed to inhibit the expression of β-site APP cleaving enzyme 1 (BACE1) and the phosphorylation levels of PKR_Thr446_ and eIF2α_Ser51_ by upregulating the expression of SOD, CAT in mice mutant for overexpression of amyloid precursor protein gene (Zhang et al., 2016). Oxidative stress-induced phosphorylation of eIF2α_Ser51_ has been found to increase BACE1 translation and promote Aβ production (O'Connor et al., 2008). These findings suggested that GAS may diminish Aβ production by curbing the oxidative stress-mediated PKR/eIF2α pathway, thus alleviating cognitive impairment in AD mice. In addition, Fasina et al. (2022) found that 90 or 210 mg/kg of GAS inhibited the increase of pro-inflammatory factors in the brain of D-galactose-induced AD model mice and induced the proliferation of beneficial microorganisms in the intestinal tract, thus suggesting that GAS may improve memory deficits in AD model mice by attenuating neuroinflammation through the gut-brain axis. A further study used LPS-stimulated mice to replicate pathological alterations in the initial stages of AD, revealing that GAS (100 mg/kg) suppressed TLR4/TRAF6/NF-κB pathway-related proteins expression, promoted the transformation of microglia from a pro-inflammatory to an anti-inflammatory phenotype, and mitigated neuroinflammation in hippocampal neurons, thereby improving the LPS-induced deficits in memory and learning (Wang et al., 2024).

In conclusion, Aβ significantly influences the formation and development of AD. GAS exerts an anti-oxidative stress effect by inhibiting PKR/eIF2α and ERK1/2-Nrf2 pathways. The mitigating effects of GAS on AD are mediated by the TLR4/TRAF6/NF-κB pathway, which involves gut microbes and microglia activation. GAS has a crucial role in AD-induced neurological impairments through suppressing neuroinflammation and oxidative stress (Figure 3; Table 4).

**TABLE 4:** Summary of the targets/pathways/mechanisms and effects of gastrodin on neurodegenerative disorder.

Disease		Inducer	Experimental model		Dose		Targets/mechanisms		Effects			Refs	
AD		*In vitro:* Aβ_1-42_ (10 μM)	*In vitro:* hippocampal neurons		*In vitro:* 0, 0.1, 0.3, 1, 3, 10, 30, 100, 300 μM		*In vitro:* mRNA and protein expression of SOD, Nrf2↑		Protects hippocampal neurons against Aβ (1–42)-induced neurotoxicity by activation of ERK1/2 signaling pathway			Zhao et al. (2012)	
							The content and mRNA expression of CAT↑						
							protein expression of ERK1/2, p-ERK1/2↑						
AD		*In vivo:* NA	*In vivo:* Tg2576 transgenic mice		*In vivo:* 60 mg/kg		*In vivo:* SOD, CAT↑		Inhibits BACE1 expression under oxidative stress conditions by inhibition of the PKR/eIF2α signaling pathway			Zhang et al. (2016)	
							MDA, BACE1↓						
							The ratio of p-PKR_Thr446_/PKR, p-eIF2α_Ser51_/eIF2α↓						
		*In vitro: NA*	*In vitro:* SH-SY5Y cells		*In vitro:* 25, 50 μM		*In vitro:* P-PKR_Thr446_, P-eIF2α_Ser51_↓						
AD		*In vitro:* D-galactose	*In vivo:* mice		*In vivo:* 3, 90, 210 mg/kg (i.g.)		*In vivo:* mRNA expression of BDNF↑		Alleviates neuron inflammation of the AD mouse model via partly targeting the microbiota–gut–brain axis			Fasina et al. (2022)	
							protein expression of TLR4, p-IκBα↓, LPS, IL-1β, TNF-α, IL-6↓						
							ZO-1, occludin↑						
AD		*In vivo:* LPS (2 mg/kg)	*In vivo:* C57BL/6 mice		*In vivo:* 100 mg/kg (i.p. injection)		*In vivo:* protein expression of p-Stat3, p-NF-κBp65, TLR4, TRAF6↓		Alleviates neuroinflammation in AD model mice through inhibition of the TLR4/TRAF6/NF-κB signaling pathway and activation of microglia and astrocytes			Wang et al. (2024)	
							levels of Iba1, GFAP↓						
							mRNA and protein expression of TNF-α, IL-1β↓						
							mRNA of Arg-1↑						
AD		*In vitro:* LPS (1 μg/mL)	*In vitro:* BV-2 cells		*In vitro:* 1, 10 μg/mL		*In vitro:* iNOS, CD206↑					Wang et al. (2024)	
							p-Stat3, NF-κBp65↓						
PD		*In vivo:* rotenone (2 mg/kg/d)	*In vivo:* Wistar rats		*In vivo:* 0.2 g/kg/d (i.g.)		*In vivo:* TH↑		Exerts a protective effect on dopaminergic neurons by reducing the expression of pro-inflammatory factors			Li et al. (2012)	
							TNF-α, IL-1β, IL-6↓						
PD		*In vivo:* MPTP (30 mg/kg/day)	*In vivo:* C57BL/6 mice		*In vivo:* 10, 30, 60 mg/kg (i.g.)		*In vivo:* TH, SOD↑		Restores TH levels and GFAP expression and protects dopamine neurons from neurotoxicity			Kumar et al. (2013)	
							GFAP, cleavage PARP, caspase-3↓						
							mRNA expression of Bcl-2↑						
							mRNA expression of Bax and the ratio of Bax/Bcl-2↓						
		*In vitro:* MPTP (30 mg/kg/day)	*In vitro:* SH-SY5Y cells		*In vitro:* 1, 5, 25 μM		*In vitro:* cleavage PARP, ROS↓						
							SOD↑						
							mRNA expression of HO-1, Bax, Bax/Bcl-2↓						
							mRNA expression of Bcl-2↑						
PD		*In vivo:* MPTP	*In vivo:* C57BL/6 mice		*In vivo:* 60 mg/kg (i.p. injection)		*In vivo:* mRNA and protein expression of HO-1, GSH, SOD, Nrf2↑		Activates the ERK1/2 signaling pathway and promotes nuclear translocation of Nrf2 to play an antioxidant role			Wang et al. (2014)	
							protein expression of p-ERK1/2↑						
							protein expression of MDA↓						
PD		*In vivo:* 6-OHDA (8 μg/2 μL/rat)	*In vivo:* Wistar rats		*In vivo:* 20, 40, 80 μg/3 μL/rat		*In vivo:* The activity of MPO↓		Reduces peroxidase activity, lipid peroxidation levels, NO production, and restores TAC levels to alleviate PD.			Haddadi et al. (2018)	
							level of MDA, NO↓						

AD, Alzheimer’s disease; Aβ, beta-amyloid; SOD, superoxide dismutase; Nrf2, nuclear factor erythroid 2-related factor 2; CAT, catalase; ERK, extracellular regulated kinase; NA, not applicable; MDA, malondialdehyde; BACE1, β-site APP, cleaving enzyme 1; PKR, protein kinase; eIF2α, eukaryotic initiation factor-2a; BDNF, brain-derived neurotrophic factor; TLR4, toll-like receptor 4; IκBα, Inhibitor of kappa B-α; LPS, lipopolysaccharide; IL, interleukin; TNF-α, tumor necrosis factor-α; ZO-1, zonulin-1; Stat3, signal transducer and activator of transcription 3; NF-κB, nuclear factor-κB; TRAF6, tumor necrosis factor receptor-associated factor 6; GFAP, glial fibrillary acidic protein; Arg-1, arginase-1; iNOS, inducible nitric oxide synthase; PD, Parkinson’s disease; TH, tyrosine hydroxylase; MPTP, 1-methyl-4-phenyl-1, 2,3,6-tetrahydropyridine; PARP, poly ADP-ribose polymerase; Bax, Bcl-2-associated X protein; Bcl-2, B-cell lymphoma-2; 6-OHDA, 6-hydroxydopamine; GSH, glutathione; MPO, myeloperoxidase; SD, Sprague-Dawley; NO, nitric oxide.

#### 5.2.2 Improvement of Parkinson’s disease (PD)

PD is the second most common neurodegenerative disorder, whose principal symptoms encompass a spectrum of motor manifestations, including rigidity, tremor, and bradykinesia (Moustafa et al., 2016). Although the etiology of PD has not been clearly defined, epidemiological and genetic studies have indicated that dopamine neuron deficiency, neuroinflammation, and oxidative stress characterize its pathogenesis (Ye et al., 2023).

Kumar et al. (2013) investigated the influence of GAS on dopamine neurons using a PD mouse model induced by 1-methyl-4-phenyl-1,2,5,6-tetrahydropyridine (MPTP). They found that a dose of 5 and 25 μM of GAS led to a restoration of glial fibrillary acidic protein (GFAP) expression and a recovery of tyrosine hydroxylase (TH) levels in a dose-dependent manner. They also noted that GAS treatment could protect dopamine neurons from neurotoxicity. A further fisetin-induced PD rat model verified the Kumar’ study data, confirming that GAS exerts a protective effect on dopaminergic neurons by decreasing the expression of pro-inflammatory factors (Li et al., 2012). Moreover, Wang et al. (2014) demonstrated that GAS exerts antioxidant effects in mitigating dyskinesia in MPTP-induced PD model mice through the promotion of Nrf2 nuclear translocation and the activation of the ERK1/2 pathway. Similarly, the same findings were obtained in a 6-hydroxydopamine (OHDA)-induced PD mouse model, which demonstrated that GAS significantly inhibited the severity of catalepsy in PD mice by reducing nitric oxide (NO) production, peroxidase activity and lipid peroxidation levels and restoring total antioxidant capacity levels (Haddadi et al., 2018).

According to the above research, the protective effect of dopamine neurons may represent a crucial mechanism for treating PD by GAS. Nrf2 are significant action targets, and ERK1/2-Nrf2 pathways are essential for GAS to exert anti-inflammatory and antioxidant effects and attenuate dopamine neuron damage and apoptosis (Figure 3; Table 4).

### 5.3 Effects of GAS on episodic neurological diseases

#### 5.3.1 Anti-epileptic effects

Epilepsy is a seizure disorder of the nervous system featuring unprovoked seizures and recurrent (Devinsky et al., 2018). Seizures occur due to hypersynchronous abnormal, excessive discharges of neurons in the brain, causing a paroxysmal change in neural function (Stafstrom and Carmant, 2015).

In a study conducted by Chen et al. (2017), pentylenetetrazole (PTZ)-induced seizure mice were administered GAS, which was found to mitigate PTZ-induced microglia activation through the inhibition of the MAPK pathway, cyclic adenosine monophosphate-responsive element binding protein (CREB) phosphorylation, and NF-κB, accompanied by a reduction in TNF-α and IL-1β expression. Additionally, GAS demonstrated the ability to attenuate neuroinflammation within the mouse brain and abnormal synchronous discharges while reducing seizure intensity and prolonging seizure duration. The same drug induction was used by Jin et al. (2018), who discovered that GAS (600, 800, and 1,000 µM) reinforced the antioxidant defense system by inhibiting the production of reactive oxygen species (ROS) and enhancing the activity of SOD and CAT. It was also found that this may assist in protecting zebrafish from further seizures. The mRNA levels of CAT, Mn-SOD, Cu/Zn-SOD, and glutathione peroxidase 1a in PTZ-induced animals were nearly normal at a concentration of 1,000 µM of GAS. GAS attenuated PTZ-induced oxidative stress and alleviated seizures in a concentration-dependent manner. In their study, Yang et al. (2021) used the temporal lobe epilepsy model induced by lithium-pilocarpine, finding that GAS (50 mg/kg) attenuated seizure severity and neuronal excitability by enhancing the γ-aminobutyric acid (GABA) A receptor.

**FIGURE 4:**
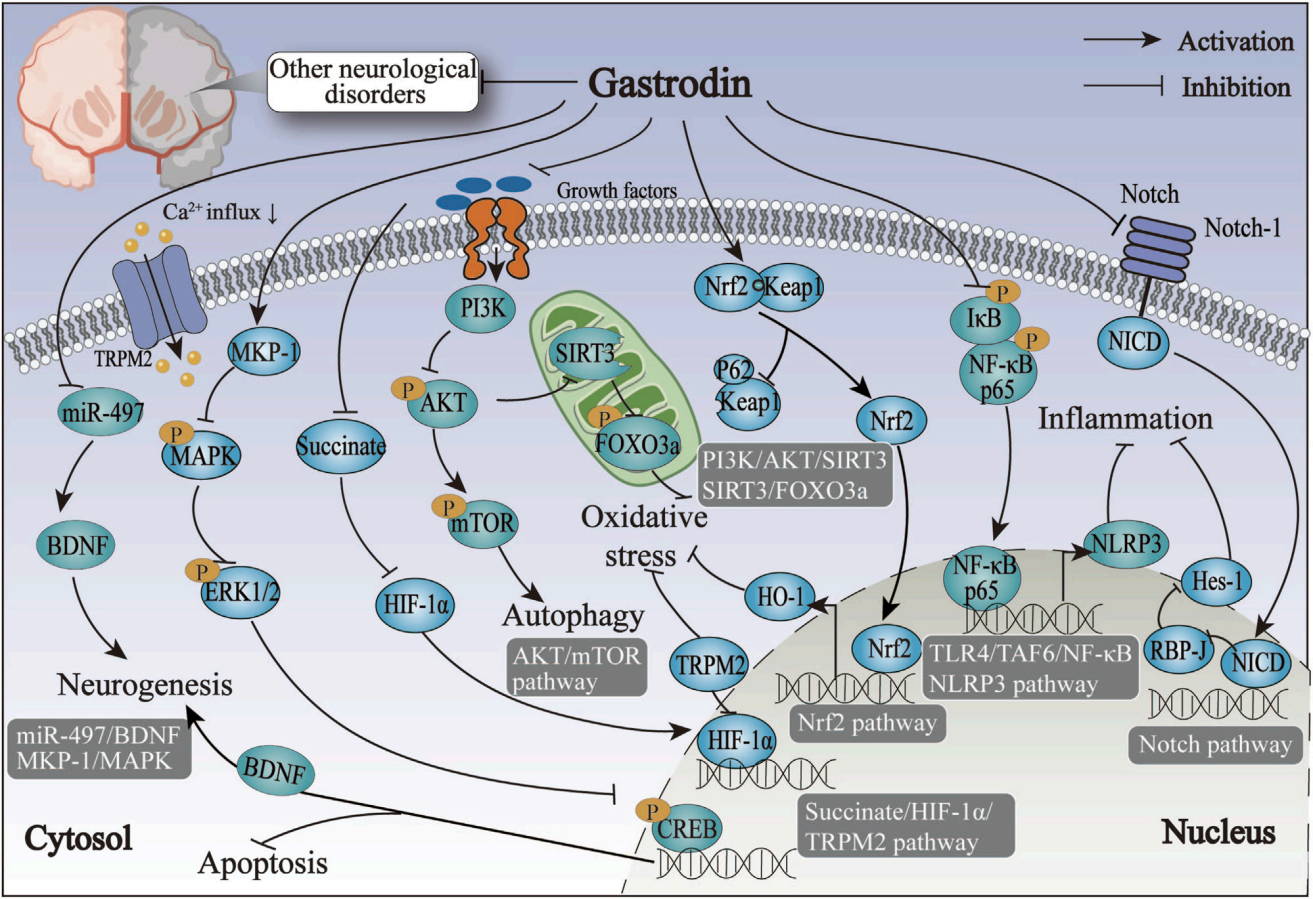
The therapeutic mechanism and critical pathways of gastrodin against epileptic neurological disorder, peripheral nerve injury, traumatic brain injury, hypoxic-ischemic brain damage, depression, and nervous system tumors.

The above overview suggests that MAPK and GABA may be involved in neurotoxicity-induced seizures, such as PTZ-induced and lithium-pilocarpine, through the immune response pathway. Furthermore, GAS may exert its antiepileptic properties by inhibiting inflammatory and oxidant reactions via the MAPK and GABA pathway. However, future *in vitro* and clinical trials are needed to further investigate the specific effects of GAS on other aspects of GABA metabolism (Figure 4; Table 5).

**TABLE 5:** Summary of the targets/pathways/mechanisms and effects of gastrodin on epileptic neurological disorder.

Disease	Inducer	Experimental model	Dose	Targets/mechanisms	Effects	Refs
Epilepsy	*In vivo:* PTZ (5 mM)	*In vivo:* Zebrafish (*Danio rerio*)	*In vivo:* 600, 800, 1,000 μM	*In vivo:* mRNA expression of c-fos, Keap1↓	Inhibits ROS generation and enhances the antioxidant defense system, and increases SOD and CAT activities	Jin et al. (2018)
				mRNA expression of Mn-sod, Cu/Zn-sod, CAT, Gpx1a, Nrf2↑		
				content of ROS, MDA↓		
				activities of SOD, CAT↑		
Epilepsy	*In vivo:* PTZ (90 mg/kg)	*In vivo:* C57BL/6 mice	*In vivo:* 50, 100, 200 mg/kg	*In vivo:* IL-1β, TNF-α, p-ERK1/2, p-JNK, p-p38, CREB, IκB-α↓	Reduces neuroinflammation and abnormal synchronous discharges by inhibiting the MAPK signaling pathway and IκB-α and CREB phosphorylation	Chen et al. (2017)
				IL-10, p-MKP-1↑		
Epilepsy	*In vivo:*LC (3 mEq/kg), Pamine (25 mg/kg)	*In vivo:* SD rats	*In vivo:* 50 mg/kg (i.p. injection)	*In vivo:* GABA_A_ receptor α1↑	Alleviates seizure severity and neuronal excitability by enhancing the transmission of GABA_A_ receptors	Yang et al. (2021)
Migraine	*In vivo:* NA	*In vivo:* SD rats	*In vivo:* 30, 100 mg/kg (i.v.)	*In vivo:* NA	Inhibits nociceptive dural-evoked neuronal firing in the trigeminocervical complex	Zhao et al. (2018)
Migraine	*In vivo:* NTG (10 mg/kg)	*In vivo:* C57BL/6 mice	*In vivo:* 200 mg/kg (i.p. injection)	*In vivo:* ROS, HIF-1α↓	Inhibits TRPM2-dependent Ca^2+^ inward flow by attenuating succinate accumulation and HIF-1α-associated transcriptional regulation, and thereby alleviation of trigeminal neuron cell death and neurotoxicity	Ma et al. (2024)
				mRNA expression of IL-1β, IL-6, TNF-α↓		
				mRNA and protein expression of Trpm2↓		
				succinate, Ca^2+^ influx↓		

PTZ, pentylenetetrazole; Keap1, kelch like ECH-associated protein 1; Mn-sod, manganese superoxide dismutase; Cu/Zn-sod, copper/zinc superoxide dis mutase; CAT, catalase; Gpx1a, glutathione peroxidase 1a; Nrf2, nuclear erythroid factor 2-related factor 2; ROS, reactive oxygen species; MDA, malondialdehyde; SOD, super oxide dismutase; IL, interleukin; TNF, tumor necrosis factor; ERK1/2, extracellular regulated kinase 1/2; JNK, c-Jun N-terminal kinases; CREB, cyclic adenosine monophosphate-responsive element binding protein; IκB-α, inhibitor of kappa B-α; MKP-1, mitogen-activated protein kinase phosphatase-1; LC, methyl scopolamine; Pamine, methylscopolamine; SD, Sprague-Dawley; GABA_A_, γ-aminobutyric acid A; NA, not applicable; NTG, nitroglycerin; HIF-1α, hypoxia inducible factor-1α; Trpm 2, transient receptor potential melastatin 2.

#### 5.3.2 Analgesic effects of migraine

Migraine is a pervasive episodic neurological disorder (Gbd et al., 2018). The pathogenesis of migraine attacks remains incompletely understood. Modern research suggests that migraine attacks primarily relate to the trigeminovascular system, consisting of the trigeminal nerve and the intracranial vasculature projected by trigeminal nerve axons (Ashina, 2020).

Zhao et al. (2018) observed that GAS at doses of 30 and 100 mg/kg significantly inhibits dural stimulation-induced pain-related neuronal discharges in the trigeminal cervical complex and that low doses of GAS produced inhibition of persistent spontaneous nerve discharges. Ma et al. (2024) further showed that 200 mg/kg GAS impedes the succinate/hypoxia-inducible factor (HIF)-1α/transient receptor potential melastatin2 (TRPM2) pathway by regulating metabolic inhibition, which leads to a reduction in trigeminal ganglion neuron-dependent Ca^2+^influx after succinate injury and attenuates nitroglycerin-induced migraine-induced trigeminal cell death and neurotoxicity.

From the above summary, our findings suggest that GAS may possess analgesic properties that could potentially alleviate migraine symptoms by inhibiting spontaneous nerve discharges in the trigeminal ganglion. This may be achieved by reducing neurotoxicity and neuronal apoptosis via the succinate/HIF-1α/TRPM2 pathway. Nevertheless, the precise molecular mechanisms remain elusive. Future studies should focus on the mechanisms of migraines and the analgesic mechanism of GAS in treating migraines (Figure 4; Table 5).

### 5.4 Effects of GAS on nerve regeneration post PNI

PNI is a sensory and motor dysfunction resulting from acute compression or trauma to the peripheral nerves (Hussain et al., 2020). Schwann cells (SCs) are responsible for the production and secretion of neurotrophic and nerve regeneration factors, which are the primary regulators of peripheral nerve development (Jessen and Mirsky, 1999; Raphael et al., 2010).

Zuo et al. (2016) demonstrated that GAS induced the metabolism and proliferation of SCs by activating Akt phosphorylation and inhibiting ERK1/2 phosphorylation and that 200 μM was the optimal concentration of GAS for stimulating the proliferation of RSC96 SCs. Their study also revealed that GAS significantly upregulated the gene expression of neurotrophic factors, which reconfirmed that GAS exerts a neurodegenerative role by regulating SCs (Zuo et al., 2016). An *In vivo* study showed that treatment with GAS (20 mg/kg/d) resulted in a significant increase in the sciatic nerve function index and a reduction in muscle atrophy in rats with a PNI model established by sciatic nerve injury. The expression of myelin basic protein and neurofilament-200 was increased in the sciatic nerve of GAS-treated PNI rats in comparison to the control group (Li et al., 2022), which may be because GAS accelerates peripheral nerve axon growth, myelin formation, and functional recovery by upregulating the activities of SOD, CAT, and GSH in SCs through the miR-497/brain-derived neurotrophic factor (BDNF) pathway (Li et al., 2022).

In conclusion, nerve regeneration is a principal mechanism in treating peripheral nerve injury with GAS. GAS exerts its effects primarily by regulating the miR-497/BDNF and ERK1/2 pathways, with SCs pivotal in promoting peripheral nerve regeneration (Figure 4; Table 6).

**TABLE 6:** Summary of the targets/pathways/mechanisms and effects of gastrodin on peripheral nerve injury, traumatic brain injury, and hypoxic-ischemic brain damage.

Disease	Inducer	Experimental model	Dose	Targets/mechanisms	Effects	Refs.
PNI	*In vivo:* NA	*In vivo*: SD rats	*In vivo*: 20 mg/kg (i.p. injection)	*In vivo*: SOD, CAT, GSH↑	Alleviates the oxidative stress of SCs and accelerates the axonal growth, myelination, and functional recovery of peripheral nerve after PNI via miR-497/BDNF pathway	Yongguang et al. (2022)
				content of MDA↓		
				mRNA and protein expression of BDNF↑, miR-497↓		
	*In vitro:* NA	*In vitro*: RSC96, HEK293T cells	*In vitro*: 200 μg/mL	*In vitro*: SOD, CAT, GSH↑		
				content of MDA↓		
PNI	*In vitro:* NA	*In vitro*: RSC96 SC cells	*In vitro*: 0, 50, 100, 200 μM	*In vitro*: mRNA expression of GDNF, BDNF, CNTF↑	Affects SC metabolism through the activation of the PI3K pathway and inhibition of the MAPK pathway	Zuo et al. (2016)
				protein expression of p-Akt↑, p-ERK1/2↓		
TBI	*In vivo:* NA	*In vivo*: SD rats	*In vivo*: 50, 100 mg/kg	*In vivo*: protein expression of Bcl-2, Nrf2, HO-1, NQO-1, SOD, GSH-px, CAT↑	Activates the Nrf2 signaling pathway, increasing the levels of antioxidant enzymes to enhance antioxidant capacity	Wang and Dong (2021)
				Bax, cleaved-caspase-3, MDA↓		
TBI	*In vivo:* NA	*In vivo*: NA	*In vivo*: 15, 30, 60 mg/kg (i.p. injection)	*In vivo*: TNF-α, IL-1β, IL-18, GSDMD, caspase-1, caspase-11, NLRP3, ASC↓	Attenuates neurological injury in TBI rats by inhibiting the NLRP3 inflammasome signaling pathway and decreasing the level of pyroptosis	Yang et al. (2022)
HIBD	*In vitro*: NA	*In vivo*: SD rats	*In vivo*: 100 mg/kg (i.p. injection)	*In vivo*: Notch-1, NICD, Hes-1, RBP-JK↓	Inhibits overactivated microglial cells to reduce neuroinflammation by decreasing the expression of key components of the Notch signaling pathway	Guo et al. (2021)
				Sirt3↑		
	*In vitro*: LPS (1 μg/mL)	*In vitro*: BV-2 cells	*In vitro*: 0.17 mM, 0.34 mM	*In vitro*: Notch-1, NICD, Hes-1, RBP-JK, iNOS, TNF-α↓		
				Sirt3↑		
HIBD	*In vivo*: NA	*In vivo*: C57BL/6J mice	*In vivo*: 300 mg/kg (i.p. injection)	*In vivo*: P-PI3K, P-AKT, TGF-β1, Arg-1, CD206↑	Reduces activated microglia apoptosis through the PI3K/AKT signaling pathway	Zuo et al. (2023)
				TNF-α, Bax/Bcl-2, CD16/32↓		
	*In vitro*: LPS (1 μg/mL)	*In vitro*: BV-2 cells	*In vitro*: 0.17mM, 0.34 mM	*In vitro*: TGF-β1, Arg-1, CD206, IL-10, P-FOXO3a, P-PI3K, P-AKT↑		
				TNF-α, Bax/Bcl-2, CD16/32, ROS↓		
HIBD	*In vitro*: NA	*In vitro*: TNC1 cells	*In vitro*: 0.17, 0.34, 0.51, 0.68, 0.85, 1.02 mM	*In vitro*: IGF-1, BDNF, Sirt3↑	Inhibits the expression of pro-inflammatory factors and promotes the expression of neurotrophic factors	Zhang et al. (2021)
				TNF-α, IL-1β, Notch-1, NICD, Hes-1, RBP-JK↓		

PNI, peripheral nerve injury; NA, not applicable; SD, Sprague-Dawley; SOD, superoxide dismutase; CAT, catalase; GSH, glutathione; MDA, malondialdehyde; BDNF, brain-derived neurotrophic factor; GDNF, glial cell-derived neurotrophic factor; CNTF, ciliary neurotrophic factor; AKT, protein kinase B; TBI, traumatic brain injury; ERK, extracellular regulated kinase; Bcl-2, B-cell lymphoma-2; Nrf2, nuclear factor erythroid 2-related factor 2; HO-1, heme oxygenase-1; NQO-1, NAD (P)H quinone dehydrogenase 1; GSH, glutathione; TNF, tumor necrosis factor; IL, interleukin; GSDMD, Gasdermin D; NLRP, nod-like receptor protein; ASC, apoptosis-associated speck-like protein; iNOS, inducible nitric oxide synthase; NF-κB, nuclear factor-κB; HIBD, hypoxic-ischemic brain damage; NICD, notch intracellular domain; Hes-1, Transcription factor hairy and enhancer of split-1; RBP-JK, recombining binding protein suppressor of hairless; Sirt3, Sirtuin 3; PI3K, phosphatidylinositol-4, 5-bisphosphate 3-kinase; TGF, transforming growth factor; Arg-1, arginase-1; Bax, Bcl-2-associated X protein; IGF-1, insulin-like growth factor 1.

### 5.5 Effects of GAS on TBI neuroprotective

Traumatic brain injury (TBI), also referred to as brain damage or head injury, is defined as a head injury caused by impacts such as accidental falls and road traffic accidents (Maas et al., 2022). The principal characteristics of secondary brain damage resulting from TBI are oxidative stress and an inflammatory response (Kaur and Sharma, 2018).

After the induction of TBI in neuronal cells, the Nrf2 pathway gets activated, resulting in elevated levels of the downstream proteins NAD (P)H quinone dehydrogenase 1 and HO-1. These proteins exert neuroprotective effects. A study employing the free-fall method to construct a TBI model demonstrated that GAS (50, 100 mg/kg) protects neuronal cells from TBI-induced injury by activating the Nrf2 signaling pathway, decreasing MDA levels and increasing antioxidant enzyme levels, including GSH-peroxidase, CAT, and SOD (Wang and Dong, 2021). Another study (Yang et al., 2022) employed the same experimental method to establish a TBI model to investigate the mechanism of action of GAS in alleviating TBI injury from the perspective of inflammation. It was found that the GAS (15, 30, and 60 mg/kg, intraperitoneal injection) group could reduce the production of TNF-α, IL-1β and IL-18 and cellular death after TBI by suppressing the NLRP3 inflammasome pathway, in comparison with the TBI group. This effect was more significant when the dose of GAS was 60 mg/kg than 30 mg/kg (Yang et al., 2022), which aligns with the observations of Wang et al. and indicates that GAS has a dose-dependent impact on the management of cerebral injury in TBI.

In conclusion, the modulation of inflammatory factors, oxidative factors, Nrf2, and NLRP3 inflammasome pathways appear to have pivotal roles in regulating TBI by GAS (Figure 4; Table 6). The therapeutic effect of GAS on TBI is dose-dependent. However, further investigation is necessary to ascertain the optimal therapeutic efficacy and safety dose.

### 5.6 Effects of GAS on HIBD microglial activation

Hypoxic-ischemic brain damage (HIBD) mainly results from perinatal asphyxia or some other etiology, resulting in brain damage due to hypoxia and reduced blood perfusion to the brain, accompanied by a high mortality rate (You et al., 2023). Microglial activation is a hallmark of HIBD in neonates (Del and Becker, 1994).

*In vivo* and *in vitro* experiments conducted by Guo et al. (2021) showed that GAS exerts an inhibitory effect on the overactivation of microglia in both LPS-induced BV-2 microglia and HIBD model mice by decreasing the expression of the Notch signaling pathway and its related key proteins. Concurrently, GAS enhances the expression of Sirt3, which reduces the expression of TNF-α, and attenuates neuroinflammation. Zhang et al. (2021) found consistent results in an astrocyte model of hypoxia-ischemia established through oxygen-glucose deprivation. Their findings indicated that GAS significantly improved the pro-inflammatory environment in hypoxia-ischemia-induced TNC1 astrocytes through the Notch and Sirt3 pathways, thereby promoting the secretion of neurotrophic factors and exerting neuroprotective effects. Zuo et al. (2023) further investigated the effect of GAS on the Sirt3 pathway, finding that 300 mg/kg GAS regulated Sirt3 in microglia that 300 mg/kg of GAS regulated Sirt3 in microglia through the phosphatidylinositol-4,5-bisphosphate 3-kinase (PI3K)/AKT pathway, effectively reduced CD16/32, TNF-α levels in HIBD mice, and enhanced transfer growth factor (TGF)-β1, CD206 expression in HIBD mice. Concurrently, GAS inhibited lipopolysaccharide-induced ROS production in BV-2 microglial cells by promoting phosphorylation for forkhead box O3a (FOXO3a). This indicates that GAS may regulate microglia activation through the PI3K/AKT/Sirt3 and Sirt3/FOXO3a pathways, exerting antioxidant and anti-inflammatory effects on HIBD (Zuo et al., 2023).

The above overview indicates that the alleviation of HIBD by GAS is predominantly linked to a microglia-mediated inflammatory response. The underlying molecular mechanisms may be linked to the Notch signaling pathway and Sirt3 (Figure 4; Table 6).

### 5.7 Anti-depressant effects of GAS

Depression is a heterogenous disorder that belongs to the neurological system. It is also a major risk factor for the development of concurrent neurodegenerative disorders, including PD and dementia (Filatova et al., 2021). The current literature suggests that the mechanisms of depression are associated with neuroplasticity, cytokines, and neuro-immune processes (Cui et al., 2024; Hodes et al., 2015).

Previous studies have shown that administration of 100 and 200 mg/kg of GAS alleviated the behavioral symptoms of depression-like in a chronic unpredictable stress (CUS) rat model. This may be achieved by restoring the levels of the GFAP and up-regulating the phosphorylation of ERK1/2 and the levels of BDNF in the hippocampus (Zhang et al., 2014). *In vitro* studies have found that despite not enhancing astrocyte viability, 20 μg/mL of GAS upregulates ERK1/2 phosphorylation and BDNF levels, thereby protecting astrocytes from 72 h serum-free injury and exerting trophic neurological effects. Chen et al. (2016) further revealed that 100 mg/kg of GAS exerted an antidepressant effect in a rat model of depression induced by forced swimming experiments by regulating the expression of proteins related to cytoskeletal remodeling in the Slit-Robo pathway. This may be related to the fact that GAS acts as a caspase-3 inhibitor and attenuates neuronal cell damage in depression models by blocking caspase-3-mediated apoptosis (Pei et al., 2024). The results suggest that the neuroprotective and remodeling effects of GAS are crucial for the efficacy of antidepressant medications. In addition, some researchers examined the impact of GAS on a depression model developed by CUS from the perspective of inflammatory factors. Their findings demonstrated that GAS (200 mg/kg) reversed the augmented effects of CUS on IL-1β, NF-κB and p-iκB and attenuated depressive-like behaviors by mitigating the damage caused by pro-inflammatory cytokines to hippocampal neural stem cells (Wang et al., 2014). The capacity of GAS to mitigate neuroinflammation by fostering an Arg-1+ microglia phenotype via the Nrf2 pathway was corroborated in the lipopolysaccharide-induced depressed rat model (Zhang et al., 2023).

Above data suggests that the antidepressant effects of GAS are exerted by multiple mechanisms, including reducing neuronal apoptosis and enhancing neuroplasticity and anti-neuroinflammation (Figure 4; Table 7).

**TABLE 7:** Summary of the targets/pathways/mechanisms and effects of gastrodin on depression and nervous system tumors.

Disease	Inducer	Experimental model	Dose	Targets/mechanisms	Effects	Refs
Depression	*In vivo*: NA	*In vivo*: C57BL/6J mice	*In vivo*: 10, 20 mg/kg	*In vivo*: NE, 5-HT, DA, Bcl-2↑	Improves depression-like behaviors and nerve cell injury by inhibiting Caspase-3-mediated apoptosis	Pei et al. (2024)
				Caspase-3, Bax↓		
Depression	*In vivo*: NA	*In vivo*: SD rats	*In vivo*: 100 mg/kg (i.g.)	*In vivo*: 5-HT 1A receptor, CRMP2, PFN1↑	Regulates the expression of cytoskeleton remodeling-related protein in the Slit-Robo pathway and promotes hippocampal neuronal cell plasticity	Chen et al. (2016)
				Slit1, RhoA↓		
	*In vitro*: NA	*In vitro*: Hs 683 cells	*In vitro*: 50, 100, 100 μM	*In vitro*: Slit1↓		
Depression	*In vivo*: NA	*In vivo*: SD rats	*In vivo*: 50, 100, 200 mg/kg (i.p.)	*In vivo*: GFAP, BDNF↑	Protects astrocytes and promotes the expression of BDNF through activating ERK1/2	Zhang et al. (2014)
	*In vitro*: NA	*In vitro*: hippocampal astrocytes	*In vitro*: 5, 10, 20, 50, 100 μg/mL	*In vitro*: BDNF, p-ERK1/2↑		
Depression	*In vivo*: NA	*In vivo*: SD rats	*In vivo*: 50, 100, 200 mg/kg (i.p.)	*In vivo*: p-IκB, NF-κB, IL-1β↓	Alleviates depressive-like behavior by protecting the hippocampal neural stem cells from the damage of the pro-inflammatory cytokine IL-1β	Wang et al. (2014)
	*In vitro*: NA	*In vitro*: hippocampal NSC cells	*In vitro*: 5, 10, 20, 50 μg/mL	*In vitro*: IL-1β↓		
				The ratio of BrdU^+^/PI^+^ cells, BrdU^+^/PI^+^↑		
Depression	*In vivo:* LPS 0.25 mg/kg/d	*In vivo:* C57BL/6 mice	*In vivo:* 25, 50, 100 mg/kg/d (i.p.)	*In vivo and In vitro:* mRNA and protein expression of Nrf2, p-Nrf2↑	Attenuates neuroinflammation in LPS-induced depressed rats by promoting an Arg-1^+^ microglia phenotype through the Nrf2 pathway	Zhang et al. (2023)
	*In vitro:* LPS 5 μg/mL	*In vitro:* Primary microglia	*In vitro:* 25, 50, 100 μM	mRNA expression of Arg-1, IL-10↑		
				mRNA expression of iNOS, CD11b, CD86, NLRP3, IL-1β, IL-6↓		
Nervous system tumors	*In vivo*: NA	*In vivo*: BALB/c nude mice T98 cells	*In vivo*: 40 mg/kg	*In vivo*: HOXD10, ACSL4↑	Induces the occurrence of glioma ferroptosis by up-regulating HOXD10 and ACSL4 and down-regulating KI67 and PCNA proteins	Cao et al. (2023)
				KI67, PCNA↓		
	*In vitro*: NA	*In vitro*: HT22, C6, LO2, HK2, HPDE, NHA, U251, T98, LN229 and PC12 cells	*In vitro*: 0, 5, 10, 20 μM	*In vitro*: proliferation of the glioma cells↓		
				mRNA and protein expression of HOXD10↑		
				ROS, iron, MDA↑		
				GPX, GSH↓		
Nervous system tumors	*In vivo*: NA	*In vivo*: NOD-NCG mice	*In vivo*: NA	*In vivo*: NA	Upregulates the expression of SIP1, enhances the ability of CAR11-3 migrating to the homing bone marrow, and crosses the blood–brain barrier to the brain to fight against tumors	Huang et al. (2023)
	*In vitro*: NA	*In vitro*: U87 cells, NT, and CAR-T cells	*In vitro*: NA	*In vitro*: mRNA expression of gng8, add2↑		
				release of IFN-γ↑		
				percent of perforin, CD107a↓		
				mRNA and protein expression of S1P1↑		
Nervous system tumors	*In vitro*: NA	*In vitro*: SH-SY5Y cells	*In vitro*: 0, 1, 2, 3, 4, 5 mM	*In vitro*: cell viability↑	Exhibits an anti-autophagic effect to inhibit the METH-induced Beclin-1 protein expression via the AKT/mTOR pathway	Yang et al. (2019)
				LC3B, Beclin-1, mTOR, p-mTOR, AKT, p-AKT↓		

NE, noradrenaline; 5-HT, serotonin; DA, dopamine; Bcl-2, B-cell lymphoma-2; Bax, Bcl-2-associated X protein; CRMP, 2, dihydropyrimidinase-related protein 2; PFN1, profilin 1; RhoA, Ras homologous member A; GFAP, glial fibrillary acidic protein; BDNF, brain-derived neurotrophic factor; ERK, 1/2, extracellular regulated kinase 1/2; IκB, inhibitor of kappaB; IL, interleukin; Nrf2, nuclear erythroid factor 2-related factor 2; Arg-1, arginase-1; HOXD10, Homeobox D10; iNOS, inducible nitric oxide synthase; NLRP3,nod-like receptor protein; ACSL4, acyl-CoA, synthetase-4; ROS, reactive oxygen species; PCNA, proliferating cell nuclear antigen; GPX, glutathione peroxidase; GSH, glutathione; gng8, Guanine nucleotide binding protein (G protein) gamma 8; IFN-γ, interferon-γ; S1P1, Sphingosine1-phosphate1; LC3B, light chain 3B; mTOR, mammalian target of rapamycin; AKT, protein kinase B.

### 5.8 Anti-tumor effects of GAS

Nervous system tumors represent the most lethal form of tumor in the United States of America (Miller et al., 2021). Gliomas constitute a specific type of cranial tumor, accounting for approximately one-third of all tumors affecting the CNS and brain (Berger et al., 2022). Gliomas are aggressive and malignant tumors, particularly those found in adults, such as glioblastomas and other aggressive diffuse gliomas, accounting for 54% of all malignant cases (Miller et al., 2021).

Cao et al. (2023) discovered that GAS induced the occurrence of glioma ferroptosis by up-regulating Homeobox D10 (HOXD10) and acyl-CoA synthetase-4 and down-regulating KI67 and PCNA proteins. The silencing of HOXD10 resulted in the attenuation of the suppressive action of GAS on the proliferation of glioma cells, indicating that GAS may exert its anti-glioma effect by inducing the onset of glioma iron death through the HOXD10 pathway. In addition, a separate study on neuroblastoma revealed that GAS (2 and 4 mM) diminished the expression of autophagy-related proteins LC3B and Beclin-1 in methamphetamine-induced human dopamine neuroblastoma SH-SY5Y cells by inhibiting the AKT/mammalian target of rapamycin pathway, thereby exerting anti-autophagic effects (Yang et al., 2019).

Considering the limitations of radiotherapy and chemotherapy for gliomas and their associated adverse effects on the body, researchers have initiated investigations into novel anti-tumor therapies focusing on genes and the immune systems (Xu et al., 2021). Some clinical investigators have observed that IL-13 receptor α2 (Rα2) chimeric antigen receptor T cells elicited an anti-glioma response in patients with recurrent multifocal glioblastoma without the occurrence of treatment-related side effects (Brown et al., 2016). In their recent study, Huang et al. (2023) revealed that GAS enhances the migration of IL-13Rα2 T cells to the brain, thereby facilitating the combat of glioblastoma multiforme. Xu et al. (2022) designated the T cell as CAR11-3. Their team observed that 100 mg/kg GAS affected the expression of sphingosine1-phosphate1 (S1P1), the motility of CAR11-3, and persistently traversed the BBB to the brain, where it combatted glioblastoma (Huang et al., 2023). These findings illustrate that GAS can elicit antitumor effects through an immune response (Figure 4; Table 7).

## 6 Mechanism of action of GAS on neurological disorders

### 6.1 Anti-inflammatory effect

**FIGURE 5:**
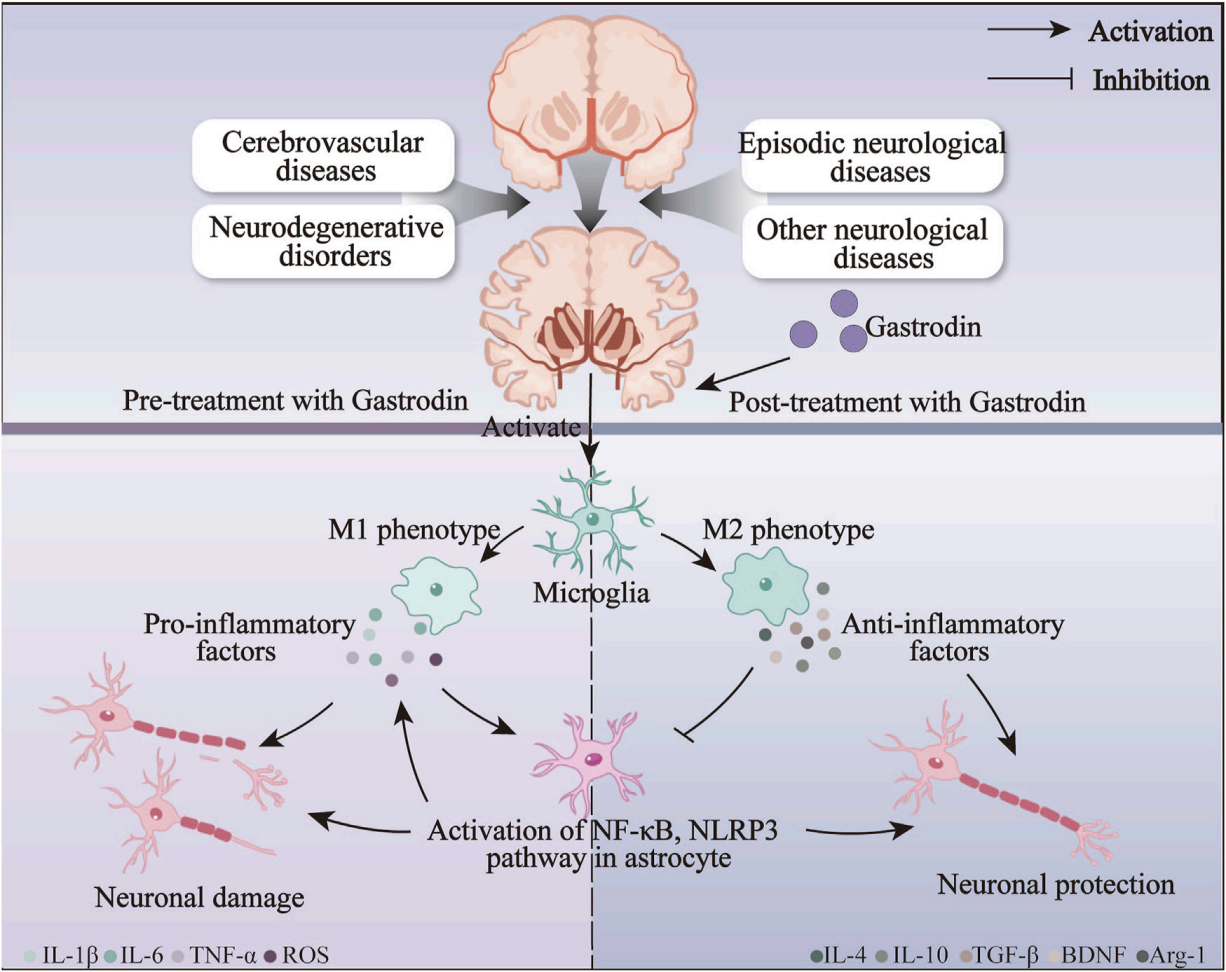
The anti-inflammatory mechanisms of gastrodin on neurological disorders via inhibiting the activation of microglia and astrocytes.

An inflammatory response is one of the most prevalent responses to neurological disorders associated with the pathophysiological processes of numerous neurological disorders (He et al., 2024). Resident immune cells in the CNS, microglia, and astrocytes are the primary glial cells implicated in the induction and regulation of inflammatory processes in neurological diseases (Colonna and Butovsky, 2017). In the event of damage to the nervous system resulting from trauma or hemorrhage, microglia are activated, thereby increasing the production of M1-type pro-inflammatory factors. This, in turn, serves to exacerbate the damage to the nerves (Shields et al., 2020). GAS can induce a transformation of microglia from the M1 pro-inflammatory phenotype to the M2 anti-inflammatory phenotype, which is achieved by the inhibition of various pathways, including those involving TLR4/TRAF6/NF-κB, PI3K/AKT, and Nrf2/STAT3, thereby reducing neuroinflammation and improving neuroinflammatory damage, and exerting an anti-inflammatory effect in neurological disorders such as AD and epilepsy. Furthermore, following the onset of neuroinflammation, activated microglia interact with astrocytes, regulating their immune response to neuroinflammation (Jha et al., 2019). The anti-inflammatory action of GAS on astrocytes is primarily achieved by inhibiting the NF-κB pathway, which prevents the assembly of NLRP3 inflammatory vesicles. At the same time, GAS also exerted neuroprotective effects by significantly improving the pro-inflammatory environment in astrocytes after brain injury through the Notch and Sirt3 pathways. The neuroprotective effect of GAS on neurological disorders may be primarily achieved by inhibiting the activation of astrocytes and microglia against neuroinflammation (Figure 5).

### 6.2 Antioxidant properties

Oxidative stress has been associated with the advancement of numerous neurological disorders, particularly neurodegenerative conditions, which are distinguished by extensive oxidative destruction of lipids, proteins, and other biological molecules (Barnham et al., 2004). In their study, Buendia et al. (2016) identified Nrf2 as a potential pivotal target in the antioxidant stress response associated with emerging neurodegenerative diseases, which is in accordance with the findings of the GAS study in treating neurological disorders. The present study has demonstrated that GAS exerts its antioxidant effects on neurodegenerative disease, primarily through the Nrf2-related pathway. GAS exerts antioxidant effects by promoting Nrf2 nuclear translocation and activating the ERK1/2 pathway, thereby reducing Aβ-induced oxidative damage in neurons and alleviating dyskinesia in the PD model mice (Wang et al., 2014). Moreover, GAS has been found to confer neuroprotective benefits against IS and TBI through the activation of the Nrf2 pathway and the enhancement of antioxidant factors production (Luo et al., 2018; Wang and Dong, 2021). This indicates that the antioxidant effects of GAS on neurological diseases are primarily mediated by the activation of Nrf2 and its associated pathways.

### 6.3 Neurotransmitter modulation

Neurotransmitters, which serve as vital messengers for transmitting messages between neurons, are crucial in the onset and development of neurological diseases and defense and treatment strategies (Teleanu et al., 2022). Amino acid neurotransmitters are a vital class of chemical messengers within the nervous system (Arumugasamy et al., 2019). Glutamate and γ-aminobutyric acid are the primary amino acid neurotransmitters within the nervous system (Dalangin et al., 2020). Maintaining equilibrium between glutamate and γ-aminobutyric acid is crucial for sustaining brain homeostasis (Wen et al., 2022). Accordingly, regulating the equilibrium between glutamate and γ-aminobutyric acid represents the critical molecular mechanism through which GAS exerts its neuroprotective effects against neurological disorders. GAS can maintain homeostatic balance in the brain and attenuate the nerve damage of seizures and HS by inhibiting glutamate excess-mediated excitatory neurotoxicity and enhancing γ-amino acid A receptor transmission. Furthermore, GAS protects dopamine neurons from neurotoxicity by restoring the expression of GFAP and TH levels, which indicates the regulatory role GAS has regarding neurotransmission.

### 6.4 Neural remodelling and neural regeneration

In neurological diseases, neuronal cells undergo apoptosis due to various factors, including trauma and hemorrhage, which occur through several pathways, including inflammation, pyroptosis, iron death, and autophagy (Moujalled et al., 2021). Consequently, remodeling and regenerating neurons are important for ameliorating brain damage in neurological diseases. Similarly, this is the principal mechanism of GAS action in treating neurological disorders. It has been demonstrated that GAS can facilitate neural remodeling and hippocampal neuron regeneration by regulating the expression of cytoskeletal remodeling-related proteins in the Slit-Robo pathway and neurotrophic factors in the PDE9/cGMP/PKG pathway, which can exert a neuroprotective role (Chen et al., 2016; Xiao et al., 2021). Furthermore, SC cells, which are glial cells in the peripheral nervous system, can produce and secret neurotrophic and regenerative factors. They also exert substantial remodeling and repair effects on peripheral nerve injuries. GAS can improve the metabolism and proliferation of SCs, thereby upregulate the expression of neurotrophic factor through the AKT, ERK1/2, and miR-497/BDNF pathways (Li et al., 2022; Zuo et al., 2016). This promotes the regeneration of peripheral neurons and the improvement of peripheral nerve injury. SCs may represent a pivotal target for GAS in the management of neurodegenerative disorders.

### 6.5 Mitochondrial function regulation

The normal functioning of the brain’s regions depends on mitochondrial energy metabolism. Dysfunctions of mitochondrial function, including Ca^2^⁺ homeostasis, cell death regulation, and mitochondrial dynamics, have emerged as a primary mechanism underlying the development of numerous neurological disorders (Cabral-Costa and Kowaltowski, 2020). The modulation of mitochondrial function represents a novel mechanism through which GAS exerts neuroprotective effects. GAS has been demonstrated to enhance mitochondrial respiration and dynamics and reverse mitochondrial dysfunction in vascular dementia by inhibiting the sirtuin 3 (Sirt3)-mediated transcription factor A acetylation pathway. Mitochondrial dysfunction resulting from altered mitochondrial dynamics in AD has been linked to Aβ accumulation (de la Cueva et al., 2022). Wu et al. (2023) demonstrated that GAS could alleviate mitochondrial respiratory depression and metabolic disturbances in VD model rats by inhibiting Aβ production. This indicates that GAS can potentially alleviate mitochondrial dysfunction by reducing Aβ accumulation, thereby protecting against Aβ-related neurological diseases.

### 6.6 Inhibition of autophagy effect

Autophagy is a precisely regulated cellular degradation pathway, and neuronal quality control is directly correlated with the physiological function of autophagy (Nikoletopoulou et al., 2015). Damage to the neuronal autophagy pathway causes neuronal degeneration, the primary factor affecting cognitive function, leading to cognitive deficits in neurological disorders such as AD and VD (Grosso et al., 2024). The regulation of autophagy has become a focal point of research in treating neurological disorders, especially neurodegenerative diseases (Corti et al., 2020; Nixon and Rubinsztein, 2024). Inhibition of neuronal autophagy represents a crucial mechanism through which GAS exerts its neuroprotective effects against neurological disorders. GAS has been demonstrated to directly inhibit the expression of autophagy-related proteins, such as LC3-II and Beclin-1, in a VD rat model. Additionally, GAS can indirectly reduce H₂O₂–induced extracellular Ca^2^⁺ inward flow in HT-22 cells by inhibiting the Ca^2^⁺/CaM-CaMKII pathway, thereby promoting the fusion of autophagic vesicles with lysosomes and the degradation of autophagic vesicles. This process maintains the homeostasis of autophagic flow, which benefits cognitive function in neurological disorders.

### 6.7 Ferroptosis bidirectional regulation

Ferroptosis represents a novel form of cell death resulting from the accumulation of iron, which generates substantial quantities of lipid peroxides. These disrupt intracellular redox homeostasis and induce cell death due to lipid peroxidation (Li et al., 2020). It has been demonstrated that glutathione peroxidase (GPX4) can exert a preventive effect against ferroptosis by catalyzing lipid peroxidation. Consequently, the GPX4 pathway has become essential for regulating the antioxidant defense against ferroptosis (Yan et al., 2021). GAS has been shown to enhance the antioxidant capacity and inhibit ferroptosis through the Nrf2/Keap1-glutathione peroxidase 4 (GPx4) pathway, thus improving cognitive impairment in rats with VD. Conversely, GAS demonstrated its anti-glioma impact by triggering the onset of glioma ferroptosis via the HOXD10 pathway. GAS exhibited antioxidant effects by inhibiting ferroptosis in non-tumor neurological disorders and anti-tumor effects by inducing ferroptosis in tumor neurological disorders, indicating that GAS may have a bi-directional modulating effect on ferroptosis in treating neurological disorders.

## 7 Clinical application of GAS in neurological disorders

### 7.1 Single-agent of GAS clinical application in neurological disorders

Currently, GAS is approved for clinical use in China by the China Drug Administration in various dosage forms, such as injection, tablet and capsule. In clinical practice, GAS injection is the most commonly used form of the drug, and it is primarily employed in the treatment of neurological disorders such as cerebral infarction, migraine, and traumatic brain injury (Table 8).

**TABLE 8:** Single-agent of gastrodin clinical application in neurological disorders.

Disease	Study type	Gastrodin form, dose and therapy	Control group	Efficacy and results	Adverse events	Refs
IS	Meta-analysis (12 RCTs)	Gastrodin injection	Compound Danshen injection	Total effective rate↑, neural functional deficit score↓	No obvious adverse reactions	Zhou (2018)
			Western medicine treatment	The levels of PV, WBV, Hct, RBC AI and FIB↓, the levels of TC, TG, LDL, HDL↓		
IS	RCT (n = 106)	Gastrodin injection, 0.6 g (i.v. gtt), once daily for 21 days	Compound Danshen injection	Remission rate (94.3%), GCS scores↑, NIHSS score↓, incidence of adverse reactions↓	Gastrointestinal symptoms, blood platelet reduce, headache	Xiu-Yun (2020)
Migraine	RCT (n = 80)	Gastrodin injection, 0.6 g (i.v.gtt), once daily for 10 days	Flunarizine capsules	Total effective rate (92.5%), VAS score↓, the levels of CGRP, NO and ET↓	NA	Yang et al. (2017)
Post-TBI Syndrome	RCT (n = 90)	Gastrodin sustained-release tablets, 150 mg, twice daily for 4 weeks	Flunarizine capsules	Total effective rate (93.33%), SCL-90 score↓, the levels of IGF-1 and IL-6↓	No obvious adverse reactions	Xuegong (2015)
		Gastrodin tablets, 100 mg, three daily for 4 weeks		Total effective rate (73.33%), the levels of IGF-1 and IL-6↓		
TBI	RCT (n = 60)	Gastrodin injection, 0.6 g, once daily for 2 weeks	Mouse nerve growth factor injection	Abnormal blood pressure, respiration and heart rate were improved; GCS and MMSE score↑, NIHSS score↓	Rash	Ming (2015)

IS, ischemic stroke; RCT, randomized controlled trial; PV, plasma viscosity; WBV, whole blood viscosity; Hct, hematocrit; RBC AI, red blood cell aggregation index; FIB, fibrinogen; TC, total cholesterol; TG, triglycerides; LDL, low-density lipoprotein; HDL, high-density lipoprotein; GCS, glasgow coma scale; NIHSS, national institutes of health stroke scale; VAS, visual analogue scale; CGRP, calcitonin gene-related peptide; NO, nitric oxide; ET, endothelin; NA, not applicable; TBI, traumatic brain injury; SCL-90, Symptom Checklist-90; IGF, insulin-like growth factor; IL, interleukin; MMSE, Mini-Mental State Examination.

Zhou (2018) conducted a Meta-analysis of clinical studies on GAS injection for IS and screened 12 randomised controlled trials. The results showed that the improvement of nerve function, blood rheological indexes and blood lipid content of GAS injection was superior to that of Compound Danshen injection. It also demonstrated that 11 of the 12 studies lacked records of adverse reactions, and one result indicated the absence of significant adverse reactions following the administration of the drug (Zhou, 2018). Xiu-Yun (2020) evaluated the efficacy and incidence of adverse reactions associated with GAS injection in a cohort of 106 patients with IS. The analysis revealed that the incidence of adverse reactions such as gastrointestinal reactions and headache was 3.8% in the GAS injection group, while it was 18.9% in the Compound Danshen injection group, which was a statistically significant difference. These findings are inconsistent with those of the previous study. Further investigation is required to ascertain whether the use of GAS has a positive effect on the incidence of adverse reactions.

In addition, another study (Yang et al., 2017) showed that the same dosage of GAS injection was capable of reducing the expression of calcitonin gene-related peptide, NO and endothelin-1 in the peripheral blood of migraine patients. It suggests that GAS may regulate vasoactive factors and inflammatory responses by modulating the vasoactive factors and inflammatory responses. Xuegong (2015) found that both GAS extended-release tablets and GAS tablets were efficacious in reducing reduce serum insulin-like growth factor-1 and IL-6 levels in patients with post-traumatic brain syndrome. In contrast, the control group was administered flunarizine hydrochloride capsules, which demonstrated no significant change. This finding corroborates the anti-inflammatory effect of GAS on neurological diseases.

### 7.2 Drug combinations of GAS clinical application in neurological disorders

In clinical practice, the combination of GAS is more prevalent than GAS alone (Table 9). Li (2016) conducted a randomised controlled trial of 100 cases of migraine patients, the results found that the clinical efficacy of GAS combined with sodium valproate was significantly higher than that of the GAS alone group. And the frequency of headache attacks, duration of attacks, headache degree, and memory of the incidence of adverse reactions were all significantly lower than those observed in the GAS alone group. In a further clinical investigation into the treatment of migraine (Zhang, 2020), it was demonstrated that the combination of GAS and nimodipine was capable of effectively reducing the mean blood flow velocity of the anterior and middle cerebral arteries in patients with migraine. Zhang and Li, 2019 obtained the same results when treating migraine with a combination of GAS and lomerizine, indicating that the association of GAS with calcium channel blockers may exert analgesic effects on migraine by enhancing blood flow velocity.

**TABLE 9:** Drug combinations of gastrodin clinical application in neurological disorders.

Disease	Study type	Gastrodin form, dose and therapy	Combination drugs and doses	Efficacy and results	Adverse events	Refs.
IS	RCT (n = 96)	Gastrodin injection, 0.6 g (i.v.gtt), once daily for 21 days	Edaravone injection, 30 mg (i.v.gtt), twice daily for 21 days	Total effective rate (89.58%), the levels of PV, WBV, Hct and FIB↓, the levels of hsCRP, Ang-Ⅱ, MCP-1and sICAM-1↓, NIHSS scores↓	NA	Feife (2018)
IS	Retrospective analysis (n = 110)	Gastrodin injection, 0.6 g (i.v.gtt), once daily for 2 weeks	butylphthalide injection, 25 mg (i.v.gtt), twice daily for 2 weeks	Total effective rate (94.74%), SSA, HAMD, HAMA and NIHSS scores↓, BI↑, the level of sTRAIL, OPG, TNF-α, and IL-8↓	Epilepsy, gastrointestinal bleeding, pulmonary infection and arrhythmia	Wang and Wang (2023)
Migraine	RCT (n = 100)	Gastrodin injection, 0.6 g (i.v.gtt), once daily for 2 weeks	Sodium Valproate, 0.2 g (i.v.gtt), three times a day for 2 weeks	Total effective rate (96%), headache attack frequency, attack duration, headache severity score↓, life quality score↑	Dizziness, limb numbness, weakness, memory decline	Li (2016)
Migraine	RCT (n = 240)	Gastrodin injection, 0.6 g (i.v.gtt), once daily for 2 weeks	Nimodipine, 60 mg (po), three times a day for 2 weeks	Total effective rate (91.67%); headache attack frequency, attack duration, headache severity score↓, the blood flow velocity of MCA and ACA↓, SF-36 score↑	Nausea, drowsiness, dizziness, diarrhea	Zhang (2020)
Migraine	RCT (n = 61)	Gastrodin capsules, 50 g (po), three times a day for 4 weeks	Toutongning capsule, 1.2 g (po), three times a day for 4 weeks	Total effective rate (93.55%); VAS score↓, SF-36 score↑, the levels of PV, Hct and FIB↓	NA	Ying (2022)
Migraine	RCT (n = 82)	Gastrodin capsules, 100 mg (po), three times a day for 8 weeks	Lomerizine, 5 mg (po), twice daily for 8 weeks	Total effective rate (95.12%), headache attack frequency, attack duration, headache severity score↓, VAS score↓, SF-36 score↑, the levels of hsCRP, HCY, LPA, VEGF and SP↓, the blood flow velocity of ACA, MCA, PCA, BA and VA↓	No obvious adverse reactions	Zhang and Li (2019)
Migraine	Meta-analysis (16 RCTs)	Gastrodin injection	Sodium valproate, dexamethasone, flunarizine, nimodipine tablet and others	Increase efficiency; reduce pain scores; improve the duration of migraine; slow down the average blood flow velocity of the middle cerebral artery	Dizziness and lethargy; dry mouth and nausea, limb numbness; weakness; memory decline, diarrhea	Zhou et al. (2022)
Trigeminal neuralgia	RCT (n = 100)	Gastrodin capsules, 100 mg (po), three times a day for 4 weeks	Phenytoin sodium, 100 mg (po), three times a day for 4 weeks	Total effective rate (94.00%), headache attack frequency, attack duration, headache severity score↓, VAS score↓, the levels of 5-HT, PGE2, IL-1β↓ and β-EP↑	Nausea and vomiting, headache, rash, dry mouth	Li Yong-Hua (2021)
Cognitive impairment after IS	RCT (n = 102)	Gastrodin injection, 0.3 g (i.m.), twice daily for 4 weeks	Oxiracetam, 0.4 g (i.m.), once daily for 4 weeks	Total effective rate (90.20%), MoCA and ADL scores↑, the levels of NSE, HCY, hs CRP↓	Skin itch, nausea, sleep disorder, mental excitation	Xiaobo (2020)
Epilepsy after stroke	RCT (n = 92)	Gastrodin tablets, 50 mg (po), three times a day for 3 months	Folate, 5 mg (po), once daily for 3 months; vitamin-B12, 25 µg (po), three times a day for 3 months	Total effective rate (95.65%), MoCA score↓, the frequency of seizures↓, the levels of HCY, HMGB-1, IL-2, IL-6↓, FOL, V-B12↑	NA	Zhou et al. (2017)
Post-traumatic syndrome	RCT (n = 78)	Gastrodin tablets, 100 mg (po), three times a day for 2 weeks	Oxiracetam injection, 4 g (i.v.gtt), once daily for 2 weeks	Total effective rate (97.44%), the scores of headaches, dizziness, anxiety, insomnia and depression↓, the levels of IL-6, IGF-1, MCP-1, sICAM-1↓	No obvious adverse reactions	Wei (2017)
Depression	RCT (n = 90)	Gastrodin injection, 0.6 g (i.v.gtt), once daily for 4 weeks	Paroxetine, 20 mg (po), once daily for 4 weeks	Total effective rate (91.1%), HAMD, SDS, SAS and SCL-90 scores↓	Nausea, headache, weakness, agrypnia, constipation, dry mouth	Di Jin (2020)

IS, ischemic stroke; RCT, randomized controlled trial; PV, plasma viscosity; WBV, whole blood viscosity; Hct, hematocrit; FIB, fibrinogen; hsCRP, high-sensitivity C-reactive protein; Ang-Ⅱ, angiotensin II; MCP-1, monocyte chemoattractant protein-1; sICAM-1, soluble intercellular adhesion molecule-1; NIHSS, national institutes of health stroke scale; NA, not applicable; SSA, Symptom Checklist-90; HAMD, hamilton depression scale; HAMA, hamilton anxiety scale; BI, barthel index; sTRAIL, soluble tumor necrosis factor related apoptosis inducing ligand; OPG, osteoprotectin; TNF-α, tumor necrosis factor-alpha; IL, interleukin; MCA, middle cerebral artery; ACA, anterior cerebral artery; SF-36, Medical Outcomes Study Short Form-36; VAS, visual analogue scale; HCY, homocysteine; LPA, lysophosphatidic acid; VEGF, vascular endothelial growth factor); SP, substance P; PCA, posterior cerebral artery; BA, bilateral artery; VA, vascular access; 5-HT, 5-hydroxy tryptamine; PGE2, Prostaglandin E2; β-EP, β-endorphin; MoCA, montreal cognitive assessment; ADL, activities of daily living; NSE, neuron specific enolase; HMGB1, High mobility group box 1; FOL, folate; V-B12, vitamin B12; IGF, insulin-like growth factor; SDS, Self-rating depression scale; SAS, Self-Rating Anxiety Scale; SCL-90, Symptom Checklist-90.

Furthermore, GAS injection combined with edaravone can reduce plasma viscosity, whole blood viscosity, haematocrit, fibrinogen, and lowered serum high-sensitivity C-reactive protein, angiotensin II, monocyte chemoattractant protein-1, and soluble intercellular adhesion molecule-1, and attenuated neurological deficits in patients with acute IS (Feife, 2018). GAS combined with olanzapine improved MoCA and ADL scores in patients with cognitive dysfunction after IS (Xiaobo, 2020), and reduced serum levels of IL-6, MCP-1 and other inflammatory factors in patients with TBI (Wei, 2017). GAS combined with folic acid and vitamin B12 significantly improves the inflammatory response while effectively controlling seizures (Zhou et al., 2017). It is suggested that the combination of GAS enhances the anti-inflammatory effect of GAS on neurological diseases.

In conclusion, GAS alone and in combination exerts protective effects on neurological diseases mainly through anti-inflammation and improving hemorheology in clinical practice. In the future, the protective mechanism of GAS on the nervous system can be explored from the perspective of vasoactive substances. In addition, the sample size of current clinical research is small, and there is a lack of high-quality, large sample and multi-center clinical research. In the future, the scientific nature of clinical research design should be strengthened.

## 8 Challenges and future perspectives

### 8.1 Challenges and limitations

One of the most significant obstacles to utilizing GAS in managing neurological diseases is the capacity to traverse the BBB. Although drug delivery systems such as AuNPs, SD, nasal ISGS, and physical enhancement methods are available, there is a paucity of clinical studies on applying GAS and its drug delivery system in neurological disorders. And the current clinical studies of GAS are characterised by small sample sizes, imperfect adverse reaction records and a predominantly observational clinical phase. There is a paucity of multi-centre, large-sample, double-blind and other high-quality clinical studies. Consequently, it is necessary to conduct additional clinical studies to validate the safety and efficacy of this approach. Secondly, the therapeutic dose of GAS varies considerably *in vivo* and *in vitro*, which may be attributable to differences in the disease model, animal species, duration of treatment, route of administration, and time point. Furthermore, GAS has been demonstrated to possess notable vasoprotective, analgesic, anticancer, and neurorestorative effects. However, the current research on GAS for neurological diseases predominantly focuses on VD, AD, and PD, with a comparatively limited investigation into cerebrovascular diseases, neuro tumours, and neurological injury categories such as peripheral nerve injury and TBI. This is particularly evident in the lack of research on peripheral neurological diseases.

### 8.2 Prospects for drug development

Currently, the clinical dosage forms of GAS are primarily injectable and oral. However, the inhalation dosage form, which can reflect the characteristics of GAS absorption through the nose, has yet to be fully explored. The development of nasal ISGS permits the administration of GAS via the naso-cerebral route, thereby circumventing the BBB and enhancing the brain targeting of GAS. It has been proposed that GAS can be used as an inhaled dosage form. Secondly, the ocular ISGS, inspired by the ocular *in situ* gel system via the eye-brain route, is also a potential drug for treating neurological disorders. Ultimately, combining GAS with ligustrazine, FA, and borneol improves the bioavailability of GAS, proposing the combined dosage form of GAS as a new idea for drug development.

### 8.3 Recommendations for future research

A review of the literature on the use of GAS in treating neurological disorders has identified several areas that warrant further investigation. Firstly, there is a need to increase the number of high-quality clinical studies related to applying GAS and its delivery system in neurological disorders. The scientific quality of the clinical study design should be improved by increasing the sample size, utilizing a multicentre approach, and implementing randomized controls. Secondly, preclinical studies should determine the safe and effective doses of GAS for treating different neurological disorders and adjust them accordingly. Thirdly, it is necessary to increase the number of studies on GAS for the treatment of cerebrovascular disease, neurogenic seizure disorders, peripheral nerve injury, and neuro-oncology, starting from the pathways of iron death, autophagy, mitochondria, ubiquitination, and acetylation. Fourthly, to improve the bioavailability of GAS, further research should focus on various nanoscale drug delivery systems, investigating the optimal effective dose of different drug delivery systems developed in different animal models of neurological disorders and their safety issues. Ultimately, the advancement of metagenomics, metabolomics, and other medical technologies is expected to further facilitate investigation into the molecular mechanisms of GAS in the treatment of neurological disorders.

## 9 Conclusion

**FIGURE 6:**
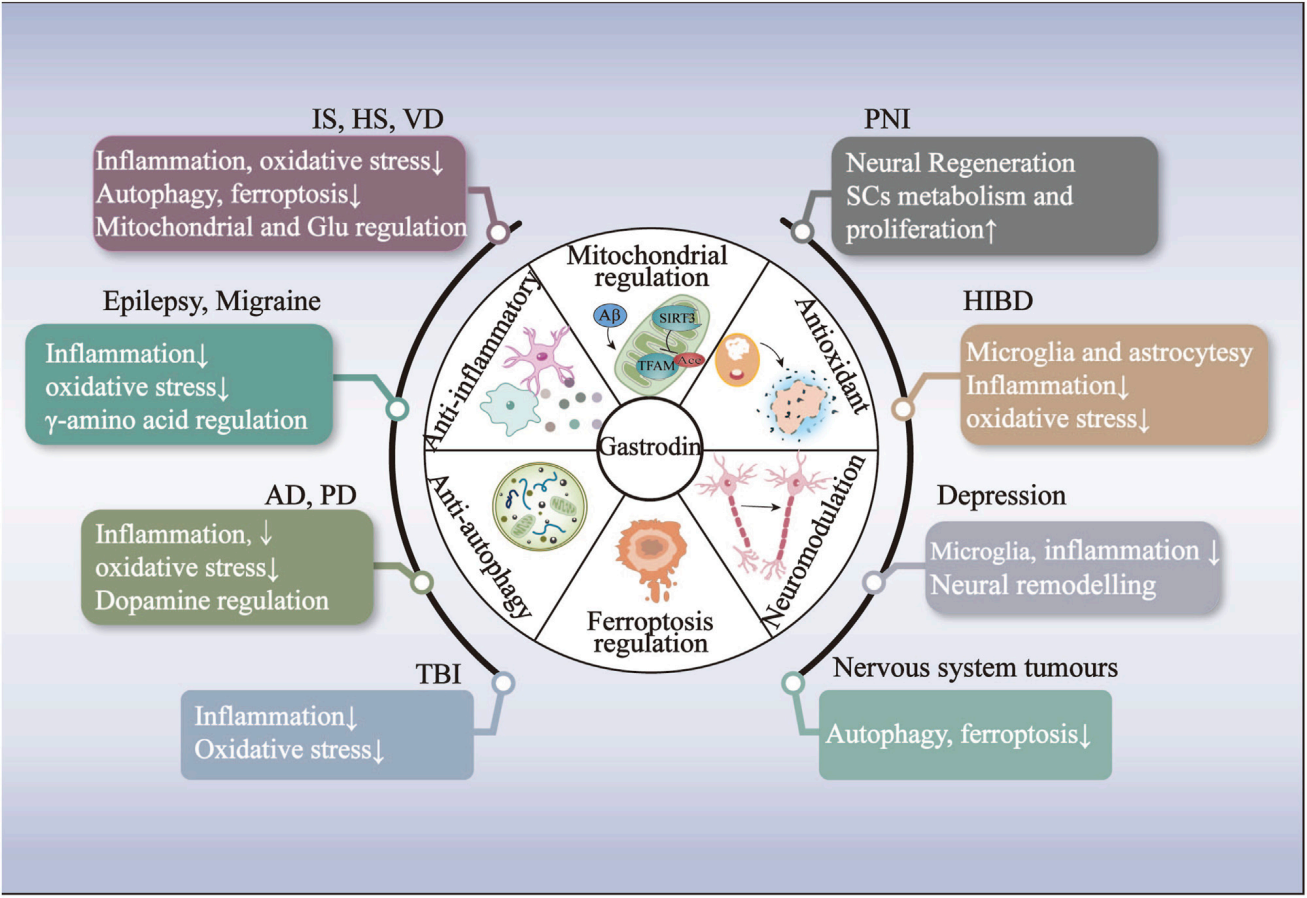
A summary of the main mechanism of gastrodin on neurological disorders.

The permeability of the BBB represents a significant challenge in the current study on the potential of GAS in treating neurological disorders. However, the development of GAS drug-carrying systems and the application of physical enhancement methods have led to a notable improvement in the bioavailability of GAS, which may have a protective role against neurological diseases through a range of mechanisms, including anti-inflammatory, antioxidant, modulation of neurological and mitochondrial functions, inhibition of autophagy, modulation of iron death and other mechanisms of action (Figure 6). This paper represents a novel perspective on the development and utilization of GAS-based drug delivery systems and the potential mechanisms underlying the action of GAS in neurological disorders. It should be noted that this review is not without limitations. In comparison to existing reviews on the use of GAS in the treatment of central nervous system disorders, this review did not include an analysis of less-studied conditions such as perioperative cognitive dysfunction, sleep deprivation, Tourette’s syndrome, and diabetic encephalopathy. However, it has expanded the scope of this review to encompass a greater number of peripheral neurological disorders. Furthermore, the synthesis and chemical structure of the GAS sources are incomplete.
